# *sic1* mutation leads to rDNA instability by partial duplication with *SIR4*

**DOI:** 10.1093/nar/gkag096

**Published:** 2026-02-10

**Authors:** Taichi Murai, Shuichi Yanagi, Yutaro Hori, Yoshio Yamamuro, Takehiko Kobayashi

**Affiliations:** Laboratory of Genome Regeneration, Institute for Quantitative Biosciences (IQB), The University of Tokyo, 1-1-1 Yayoi, Bunkyo-ku, Tokyo 113-0032, Japan; Department of Biological Sciences, Graduate School of Science, University of Tokyo, 7-3-1 Hongo, Bunkyo-ku, Tokyo 113-0033, Japan; Laboratory of Genome Regeneration, Institute for Quantitative Biosciences (IQB), The University of Tokyo, 1-1-1 Yayoi, Bunkyo-ku, Tokyo 113-0032, Japan; Laboratory of Genome Regeneration, Institute for Quantitative Biosciences (IQB), The University of Tokyo, 1-1-1 Yayoi, Bunkyo-ku, Tokyo 113-0032, Japan; Laboratory of Genome Regeneration, Institute for Quantitative Biosciences (IQB), The University of Tokyo, 1-1-1 Yayoi, Bunkyo-ku, Tokyo 113-0032, Japan; Department of Biological Sciences, Graduate School of Science, University of Tokyo, 7-3-1 Hongo, Bunkyo-ku, Tokyo 113-0033, Japan; Laboratory of Genome Regeneration, Institute for Quantitative Biosciences (IQB), The University of Tokyo, 1-1-1 Yayoi, Bunkyo-ku, Tokyo 113-0032, Japan; Department of Biological Sciences, Graduate School of Science, University of Tokyo, 7-3-1 Hongo, Bunkyo-ku, Tokyo 113-0033, Japan; Collaborative Research Institute for Innovative Microbiology, The University of Tokyo, 1-1-1 Yayoi, Bunkyo-ku, Tokyo 113-0032, Japan

## Abstract

The ribosomal RNA gene cluster (rDNA) in *Saccharomyces cerevisiae* consists of about 150 tandem copies, making it a fragile site prone to copy number changes through recombination among the repeat. While extensive research has been conducted to understand the mechanisms for rDNA stability maintenance, the relationship between the stability maintenance of rDNA and other genomic regions remains unclear. In this study, we identified a mutant, *sic1*, that exhibited instability in both rDNA and chromosome IV (chr.IV). We revealed that Ty element-mediated ectopic recombination leads to partial duplication and elongation of chr.IV. Furthermore, we found that rDNA instability is caused by an increased *SIR4* gene dosage resulting from this partial duplication. These findings suggest a link between the stability of rDNA and other genomic regions.

## Graphical Abstract

**Graphical Abstract:**
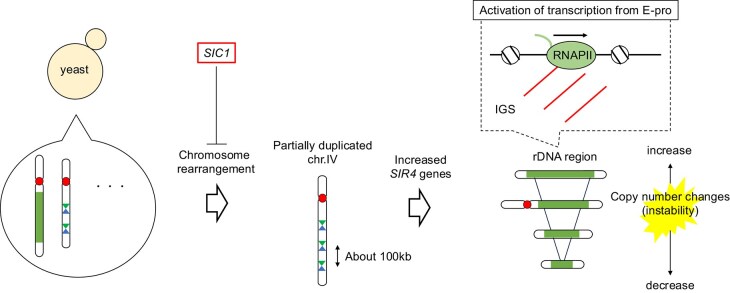

## Introduction

**Figure 1.**
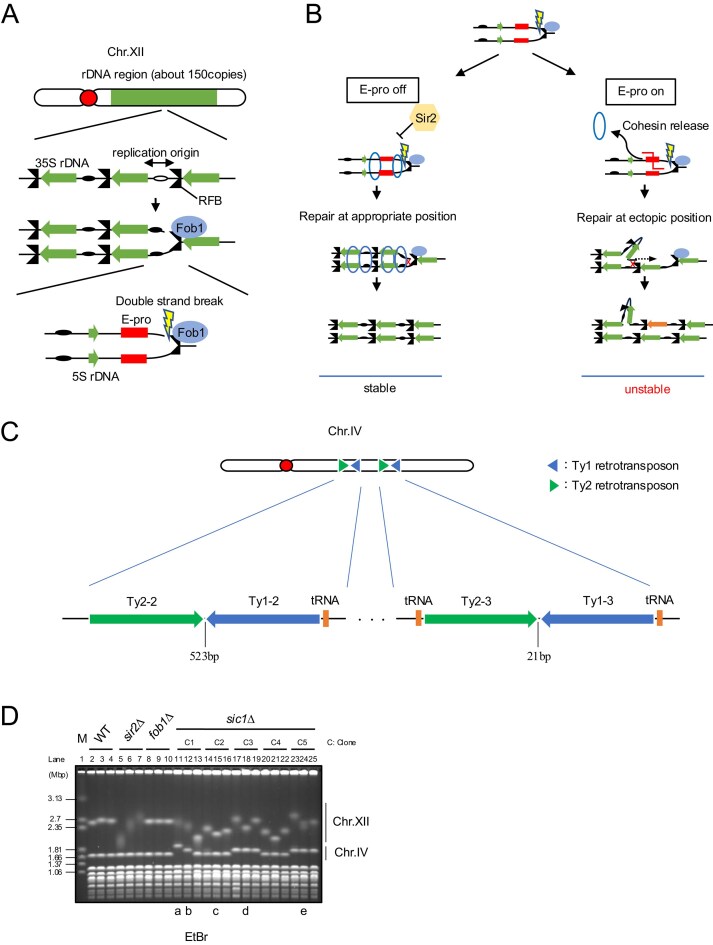
Chromosome IV (chr.IV) elongation of ∼100 kb occurs in the *sic1∆*. (**A**) Fob1-dependent replication fork arrest and DSB formation at rDNA. (**B**) Mechanism of rDNA copy number changes promoted by noncoding RNA transcription. Cohesin binding to rDNA suppresses ectopic recombination. During noncoding transcription, cohesin dissociates from rDNA, leading to unequal recombination. (**C**) Schematic diagram of chr.IV. Two pairs of Ty1 and Ty2 that are confronting each other exist at the right arm. (**D**) Pulsed-field gel electrophoresis (PFGE) analysis of *sic1Δ*. Genomic DNA of indicated strains was separated by PFGE and monitored with ethidium bromide (EtBr). Three colonies were selected from each of the five independent clones in the *sic1Δ*. *sir2Δ* and *fob1Δ* were used as controls for rDNA stability. Colonies with lowercase letters (a–e) were further analyzed.

The ribosomal RNA gene cluster (rDNA) in eukaryotes forms a highly tandem-repeated structure to supply a large amount of ribosomal RNA (rRNA). As tandem repeats tend to recombine among themselves, the rDNA is one of the most unstable regions. To ensure the stable supply of rRNA, cells have mechanisms to maintain these high copies of rDNA [1–3]. The rDNA of *Saccharomyces cerevisiae* is one of the most extensively studied. It is located on the largest chromosome, chr.XII, having about 150 rDNA units (Fig. 1A) [4]. In the rDNA of budding yeast, the fundamental mechanisms of rDNA copy number maintenance have been revealed (Fig. 1B) [5, 6]. The replication fork barrier (RFB) protein Fob1 binds to the RFB site, stalling replication fork and inducing double-strand breaks (DSBs) [7–11]. When rDNA copy number decreases by deleterious recombination between copies, transcription of noncoding RNA from the bidirectional promoter (E-pro) in the intergenic spacer (IGS) of rDNA is activated, leading to ectopic recombination by removing the sister-chromatid cohesion and copy number changes [3, 12]. However, rDNA instability has a negative effect on cells, i.e. cellular senescence [13]. In the *fob1* mutant, which has a stable rDNA due to the absence of replication fork stalling and DSB, the lifespan is much extended compared to wild type (WT) [1, 14–16]. Moreover, by depletion of Sir2 that represses E-pro transcription, rDNA become unstable and the lifespan is much shortened [17]. While these mechanisms for rDNA stabilization have been studied, the relationship between instabilities at other genomic regions is unclear.

To address this question, we have evaluated chromosome stability of rDNA and other chromosome by PFGE. In a previous study, a screening using a deletion library of about 5000 genes in budding yeast has been conducted to comprehensively investigate genes involved in the maintenance of rDNA stability [18]. In this screening, we found several mutants that had shown abnormalities in chromosome length other than chr.XII with rDNA [19, 20]. Nearly half of these mutants also exhibited rDNA instability, a proportion significantly higher than that observed overall. This suggests that these genes may play an important role in maintaining the stability of both rDNA and other genomic regions.

The most frequently observed chromosomal abnormality other than chr.XII was elongation of chr.IV, the second largest chromosome in budding yeast. Chr.IV contains the highest number of full-length yeast transposable element, Ty1 and Ty2 [21, 22]. Ty is long terminal repeat (LTR)-retrotransposons that is spread throughout the genome by a transposition mechanism [23]. There are five types of Ty, Ty1-5, and most Ty1-4 are integrated within 750 bp of the RNA pol III transcription site [transfer RNA (tRNA) genes, etc.] [21, 23]. Chr.IV has two regions where Ty1 and Ty2 are forming inverted repeats (Fig. 1C). Inverted repeats are regions of similar sequences arranged in an opposite direction. Ty2 and Ty1 show high sequence similarity [21]. These regions have previously shown to be a recombination hotspot between homologous chromosomes during mitosis [24]. This is likely due to the formation of hairpin structures between homologous sequences, which induce topological stress or stall replication forks, leading to DSBs [25].

Among the candidate mutants with abnormal size of chr.IV and unstable rDNA, this phenotype was most commonly observed in *sic1* mutants. *SIC1* is expressed from late M phase to G1 phase and regulates the cell cycle as a cyclin-dependent kinase inhibitor [26–28]. It has been suggested that in terms of contribution to genome stability maintenance, especially, inhibition of Clb5/6-cyclin-dependent kinase (CDK) activity during the G1 phase, is crucial [29]. The activity is thought to ensure sufficient time for the formation of the prereplication complex (pre-RC).

Interestingly, elongation of chr.IV was necessary for rDNA instability in the *sic1* mutant. Through the analysis of elongated region, we identified the causative gene for rDNA instability, which was *SIR4. SIR4* is known to make a complex that is required for silencing in telomere and MAT locus [30–32]. We speculate that chr.IV elongation increases the copy number of *SIR4* gene, resulting in an increased Sir4 protein level.

## Materials and methods

### Yeast strains, plasmids, primers, and culture conditions

Yeast strains and plasmids used in this study are listed in Tables S1 and S2. Yeast cells were cultured at 30°C, and cells without plasmids were cultured in Yeast extract-Peptone-Dextrose (YPD)(10 g/l yeast extract, 20 g/l peptone, and 20 g/l glucose), with 20 g/l Difco Bacto Agar added for solid medium. Plasmid-transformed strains were cultured in synthetic complete (SC) media without leucine. SC media used in this study was modified from Hartwell’ Complete media, as previously described [33]. For the construction of mutants other than those utilizing amino acid or nucleotide markers, clonNAT or G418 was added to YPD solid medium at a final concentration of 100 µg/ml and 200 µg/ml, respectively.

### Genomic DNA preparation in plugs and pulse-field gel electrophoresis

DNA plug preparation and PFGE were performed as previously described [2]. In short, 5 × 10^7^ cells were mounted in each plug, and after Zymolyase and RNaseA treatment at 37°C, proteins were degraded by proteinase K at 50°C. Plugs treated by enzymes were washed with 50 mM ethylenediaminetetraacetic acid (EDTA) (pH 7.5). The enzyme-treated plugs were cut into 3 mm-width and genome was separated using 1% agarose (Pulsed Field Certified Agarose, Bio-Rad) in 0.5× Tris-Borate-EDTA (TBE).

### Southern blotting

Southern blotting was performed as previously described [2]. On transferring DNA to the membrane following electrophoresis, the gel was soaked in 500 ml of acidic solution (0.25 M HCl) for 30 min, denatured in 500 ml of denaturation solution (1.5 M NaCl and 0.5 M NaOH) for 30 min, and neutralized in 500 ml of neutralization solution (1.5 M NaCl and 0.5 M Tris, adjust to pH 7.5 with HCl) for 30 min, all at room temperature. The DNA was then capillary transferred to Hybond-N+ (GE Healthcare) in 10× Saline-Sodium-Citrate (SSC) overnight. After transfer, DNA was fixed with 120 000 mJ/cm^2^ in a Stratalinker (Stratagene, Model 1800), and the membrane was dried under air after washing with milliQ.

The membrane was pre-hybridized in a hybridization bottle with 25 ml of hybridization buffer [10 g/l bovine serum albumin (Nacalai tesque, 01281-84), 0.5 M phosphate buffer (pH 7.2), 70 g/l sodium dodecyl sulfate (SDS), 1 mM EDTA (pH 8.0)] at 65°C for 30 min. DNA fragments amplified by polymerase chain reaction (PCR) (primer sets are listed in Table S3) served as templates for the probes, which were radioactively labeled using the Random Primer DNA Labeling Kit Ver.2. Labeled probes were purified with ProbeQuant G-50 Micro Columns (GE Healthcare), denatured at 100°C for 3 min, and added to the hybridization bottle. Hybridization was performed by incubating at 65°C overnight. After hybridization, the membrane was washed four times with wash buffer (40 mM phosphate buffer, pH 7.2, 1% SDS, 1 mM EDTA, pH 8.0) for 30 min each at 65°C. Finally, the membrane was sealed in plastic film and exposed to a storage phosphor screen, which was subsequently scanned using a Typhoon FLA7000 image (GE Healthcare).

### Flowcytometry analysis

Flowcytometry analysis was performed as previously described [2]. In brief, the saturated culture was inoculated into 50 ml of fresh YPD at a density of 3 × 10^6^ cells/ml and cultured until two divisions. 5 × 10^7^ cells were harvested by centrifugation, pellets were resuspended in 70% ethanol, and stored at −20°C. For sample preparation, 70% ethanol was removed completely, resuspended in 200 µl of 50 mM sodium citrate (pH 7.4) with 0.25 mg/ml RNaseA, and incubated at 37°C for 1 h. After RNaseA treatment, 100 µl of 50 mM sodium citrate (pH 7.4) with 0.5 mg/ml proteinase K (Nacalai) was added, and cells were incubated at 50°C for 1 h. Finally, 300 µl of 50 mM sodium citrate (pH 7.4) with 4 mg/ml propidium iodide (Sigma–Aldrich) was added. Flow cytometry analysis was performed using a BD Accuri C6 Flow Cytometer (BD Bioscience). Signals collected by FL2.A channel were plotted using R.

### Whole-genome sequence

Genomic DNA preparation was performed as previously described [2]. Whole-genome sequencing (WGS) was performed by BGI using 150 bp paired-end reads. The resulting data were aligned to the reference sequence using SNIP-O-Matic to determine the positions the reads. To identify regions with increased read depth, the number of mapped reads was calculated across 5 kb genomic bins.

### Oxford Nanopore sequencing

Genomic DNA was mildly sheared by pipetting with a P200 tip for 30 cycles. A total of 500 ng of the sheared DNA was end prepped with NEBNext Ultra II End Repair/dA-Tailing module (NEB, E7546) following the manufacturer’s protocol. The DNA was then purified using NucleoMag NGS Clean-up and Size Select (Macherey-Nagel, 744 970). Sequencing adapters were ligated to the purified DNA according to the manufacture’s protocol [Oxford Nanopore Technologies (ONT), SQK-LSK114]. The library was cleaned with NucleoMag NGS Clean-up and Size Select with Long Fragment Buffer provided in the ONT kit. One hundred nanograms of the final library was loaded onto an ONT R10.4.1 Flongle flowcell (ONT, FLO-FLG114) and sequenced. Basecalling was performed using Dorado with dna_r10.4.1_e8.2_400bps_hac@v4.3.0 model. The resulting fastq files were aligned to the reference sequences using Minimap2 (v2.24) ([xx] https://academic.oup.com/bioinformatics/article/34/18/3094/4994778). To investigate the structural variations around the putative duplicating region, we screened reads spanning >4500 bases of the region and visualized them using IGV (v2.18.4) ([xx] https://pmc.ncbi.nlm.nih.gov/articles/PMC3603213/) with “Hide small indels” setting set to 10.

### Serial dilution growth assay

Serial dilution growth assay was performed as previously described [33]. In brief, cells were inoculated from a glycerol stock into 5 ml of YPD liquid medium and grown to saturation at 30°C. The saturated cultures were adjusted to a density of 2 × 10^6^ cells/ml and then serially diluted 10-fold. Five microliters of serially diluted cell suspensions were spotted onto YPD plate medium and incubated overnight at 30°C.

### Western blotting

Western blotting was performed based on a previous study [33]. For rescue experiment using YCplac-*SIC1*-3HA, saturated culture was inoculated into 13 ml of fresh synthetic medium at a density of 3 × 10^6^ cells/ml and cultured until two divisions. 1 × 10^8^ cells were harvested, washed once with cold milliQ, and stored at −80°C. The harvested cells were resuspended in 500 µl of milliQ, 69.375 µl of 2N NaOH, and 5.625 µl of 2-mercaptoethanol, and incubated on ice for 10 min. 75 µl of 50% trichloroacetic acid was added and incubated on ice for an additional 10 min. The samples were centrifuged at 1000 rpm, 4°C, for 5 min, and the supernatant was discarded. The cells were suspended in 72 µl of 1× sample buffer (50 mM Tris–HCl, 100 mM 2-mercaptoethanol, 2% SDS, 0.017% Bromophenol blue, 5% glycerol), and boiled at 65°C for 5 min.

Polyacrylamide gel consisting of a 5% stacking gel and a 12.5% ​​running gel was poured into an electrophoresis device containing running buffer (25 mM Tris, 192 mM Glycyne, 0.1% SDS). Fifteen microliters of the prepared samples were applied and proteins were separated at 10–20 mA per gel. Proteins were transferred to immobilon-P polyvinylidene difluoride (PVDF) membrane (Merck-Millipore) in transfer buffer (25 mM Tris, 192 mM glycine, 10% vol/vol methanol) at 100 V for 1 h at 4°C using a mini-transblot cell (Bio-Rad). After transfer, the membrane was cut between the 50 and 37 kDa marker bands that comigrated with α-tubulin and Sic1-3HA, and blocked in blocking buffer (1× phosphate buffered saline, 0.05% Tween20, 5% skim milk) at room temperature for 1 h. After blocking, membranes were incubated in blocking buffers containing anti-HA tag (Santa Cruz, diluted 5 × 10^3^ fold) or anti-tubulin-horseradish peroxidase (HRP) (Bio-Rad, 10 × 10^3^ dilution) at 4°C overnight (antibodies are listed in Table S4). The membranes were washed three times 5 min with phosphate buffered saline (PBS)-T at room temperature. For Sic1-3HA detection, membrane was further incubated in blocking buffer with anti-mouse IgG-HRP (Cityva, 5 × 10^3^ dilution) at room temperature for 1 h and washed three times with PBS-T. Immobilon Western Chemiluminescent HRP Substrate (Merck Millipore) was used for chemiluminescence, and signal was captured by Fusion FL4 system (Vilber Lourmat).

### RNA preparation for RT-qPCR and northern blotting

Saturated cultures were inoculated into 50 mL of fresh YPD at a density of 3 × 10^6^ cells/ml and cultured until two divisions. 5 × 10^7^ cells were harvested, washed once with 0.1% diethylpyrocarbonate (DEPC), and stored at −80°C. RNA was extracted using Nucleospin RNA protocol (Macherey-Nagel, Item number: 740955.50).

### RT-qPCR

Reverse transcription was performed using ReverTra Ace qPCR RT Master Mix (TOYOBO). Two microliters of ReverTra Ace, 1 µl of 100 ng/µl RNA sample, and 7 µl of milliQ were mixed, and reverse transcription was performed (37° for 15 min, 50°C for 5 min, and 98°C for 5 min).

To perform qPCR, 2 µL of five-fold diluted reverse transcription product, 0.6 µl of 10 µM primer set (primers are listed in the Table S3), 5 µl of THUNDERBIRD Next (TOYOBO), and 2.4 µl of milliQ were added to a 96-well plate and suspended. qPCR was performed on a QuantStudio 1 Real-Time Instrument (Thermo Fisher Scientific) under the following conditions: predenaturation at 95°C for 30 s, followed by 40 cycles of 95°C for 5 s and 60°C for 30 s.

### Northern blotting

The samples were prepared by mixing 5 µg of RNA, 5µl of formamide (Wako), 3µl of formaldehyde (Wako), 1µl of EtBr, and 1.5µl of 10× 3-Morpholinopropanesulfonic acid (MOPS). After the samples were boiled at 65°C for 10 min, 6× loading dye (NEB) was added.

To perform electrophoresis, 1% agarose gel containing 16.7% formaldehyde (Wako) in 1× MOPS buffer was prepared, and the entire volume of each sample was applied. After electrophoresis at 25 V for 20 min at room temperature,W i.e. when the RNA entered the gel, the voltage was changed to 135 V, and further ran for 75 min.

After electrophoresis and washing the gel with 0.1% DEPC water, RNA separation was checked on a Fusion FL4 system (Vilber Lourmat). The gel was rinsed with 10× SSC and the RNA was capillary transferred to Hybond-N+ (GE Healthcare) using 10× SSC overnight. After the transfer, the RNA was fixed to the membrane with 120 000 mJ/cm^2^ in a Stratalinker 1800 (Stratagene), washed with 5× SSC and dried under air.

Hybridization was performed as the same protocol for Southern hybridization. For hybridization with the ACT1 probe, which was done after probing for IGS1-F and IGS1-R transcripts, the membranes were first stripped in boiled 0.1% SDS for 30 min, and washed with 2× SSC and 0.1% SDS.

### DSBs assay

Prepared plugs were cut to 6 mm width and washed twice with milliQ for 30 min at room temperature. The washed plugs were replaced once each with 1.5× and 1× rCutSmart Buffer (NEB) for 30 min at room temperature. A solution containing the restriction enzymes StuI and NcoI (1 unit/µl) in 1× rCutSmart buffer was added and reacted overnight at 37°C. Digested plugs arranged on a comb and DNA were separated using 0.7% agarose (STAR agarose, RIKAKEN) in 1× TBE on a Sub-cell GT electrophoresis system (Bio-Rad) in 1.5 l of 1× TBE at 2.0 V/cm for 24 h at room temperature with buffer circulation. The separated DNA was transferred from the gel to a membrane (HyBond-N+), and the signal was detected by Southern blotting.

## Results

### Chr.IV elongation occurs in *sic1* mutant

To investigate the relationship between the genomic instability of rDNA (chr.XII) and other loci, we first screened the yeast rDNA stability data base (YRSD, http://tako-lab.net/yrsd) [18] and identified 13 mutants displaying chromosome instability in both chr.XII and chr.IV. Next, we reconstructed these mutants and assessed their chr.IV elongation using PFGE. For this analysis, genomic DNA was extracted from cells streaked and cultured directly from strain stocks frozen immediately after transformation. Under these conditions, chr.IV elongation was observed exclusively in the *sic1* mutant (*sic1∆*) (Fig. 1D and Supplementary Fig. S1A). Detailed PFGE analysis of 15 *sic1∆* colonies (three colonies from each of five independent transformants) confirmed elongation in clones 1, 3, and 5. Notably, the frozen stock of clone 1 (lanes 11–13), which was derived from a single transformant colony, produced a heterogeneous population containing cells with ∼200 kbp elongation (lane 11), ∼100 kbp elongation (lane 12), and WT length chr.IV (lane 13). (Fig. 1D). In contrast, no elongation was observed in the other 12 candidate mutant strains (Supplementary Fig. S1A).

Previous studies have suggested that *SIC1* regulates the G1/S transition, ensuring sufficient time for the formation of the pre-RC [29]. Consequently, *sic1∆* mutants exhibit a phenotype characterized by a reduced G1 cell population and genomic instability. To determine whether this phenotype correlates with chr.IV elongation, selected colonies analyzed by PFGE were subjected to flow cytometry. These colonies (lanes 11, 12, 15, 18, and 24) are labeled a–e in Fig. 1D. Flow cytometry revealed that the G1 population was markedly reduced in all selected *sic1∆* colonies, regardless of the presence (a, b, d, e) or absence (c) of chr.IV elongation (Supplementary Fig. S1B). This indicates that the reduced G1 population is an intrinsic phenotype of *sic1∆*, whereas chr.IV elongation is variable.

Furthermore, to determine whether chr.IV elongation in the *sic1∆* confers a growth advantage, we performed serial dilution growth assays (Supplementary Fig. S1C). The results showed no correlation between chr.IV elongation and growth rate, suggesting that elongation is not driven by positive selection but is rather a stochastic manifestation of the genomic instability inherent to *sic1∆*.

Among the candidate mutant strains that did not exhibit chr.IV elongation were genes involved in cell cycle regulation (*DIA2* [34, 35] and *PHO85* [36] and genome integrity maintenance (*RAD18* [37] and *THP1* [38, 39]) (Supplementary Fig. S1A). The absence of chr.IV elongation in these mutants may be attributed to the limited number of generations since strain construction. It is possible that chr.IV elongation would occur in these strains following extended passaging and increased replication cycles.

### Chr.IV elongation is caused by partial duplication between Ty2–Ty1 inverted repeats in *sic1∆*

To identify the elongated region of chr.IV in *sic1Δ*, we performed WGS on *sic1Δ* colonies (a)–(e) (Fig. 1D). Genomic DNA was extracted and subjected to 150-bp paired-end sequencing to analyze read depth.

**Figure 2.**
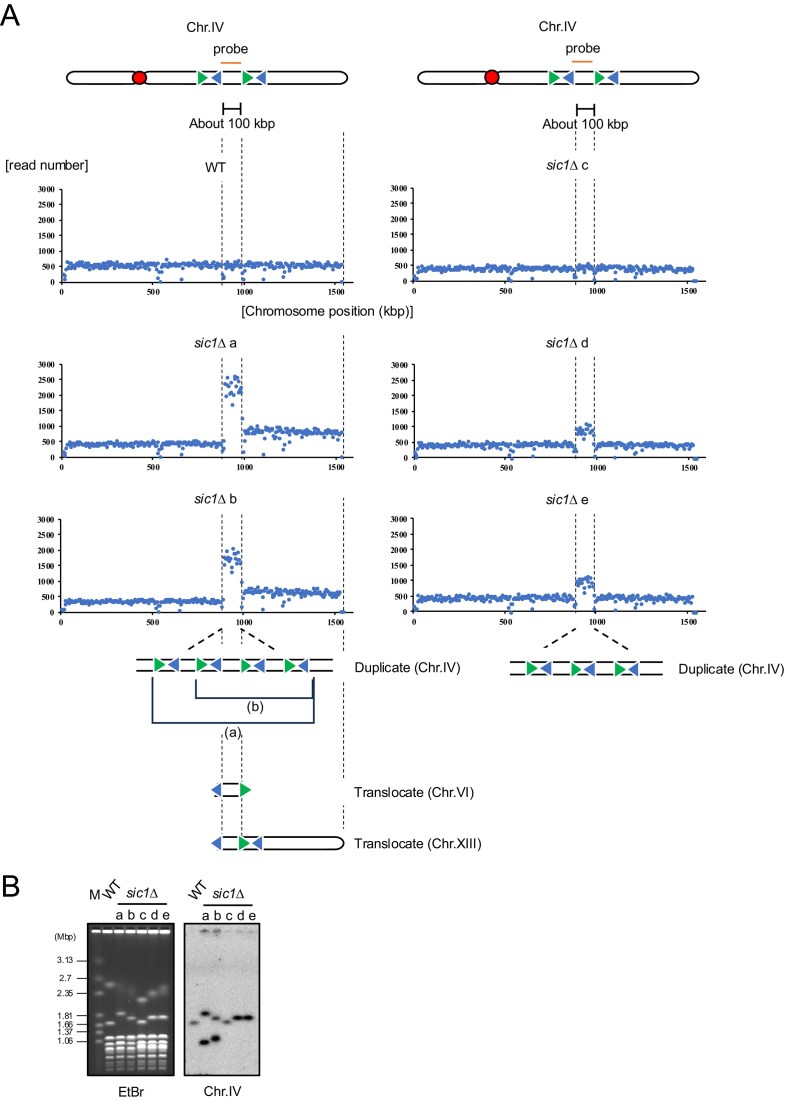
Partial duplication of chr.IV is mediated by Ty inverted repeats. (**A**) Read counts from WGS aligned to chr.IV are shown using bins of 5000 bp. The top schematic of chr.IV reflects the positions of centromere and two pairs of Ty2–Ty1 confronting each other. These positions also correspond to the *x* axis of the plots below. The bottom schematic illustrates the structure of chr.IV with chromosomal rearrangements. (**B**) Five colonies from Fig. 1D were selected for WGS, and their genomic DNA was separated by PFGE as in Fig. 1D. Total DNA was detected by EtBr. Duplicated region of chr.IV was monitored by Southern hybridization using a specific probe (Chr.IV).

Consequently, several genomic regions in *sic1Δ* mutants exhibited two- to five-fold higher coverage compared to the rest of the genome. Among these, the candidate site responsible for chr.IV elongation appeared to be a 100-kb region flanked by two sets of Ty2–Ty1 inverted repeats (Fig. 2A). Within this 100-kb region, read coverage doubled in *sic1Δ* colonies (d) and (e), whereas colony (a) exhibited a five-fold increase and (b) a four-fold increase. In colony (a), as chr.IV was elongated by around 200 kb, the region likely underwent two rounds of duplication (Fig. 2A bottom); however, the observed increase in read depth was five-fold. Additionally, in colonies (a) and (b), read depth also doubled in the region distal to the Ty2–Ty1 inverted repeats. These results suggested that the partial duplicated chr.IV segment might have translocated to another chromosome. To test this possibility, we designed a probe for the duplicated region (Fig. 2A) and performed Southern hybridization (Fig. 2B). As expected, signals were detected not only on chr.IV but also on a different chromosome. When Southern hybridization was also performed against the gel from Fig. 1D with this probe, signals distinct from chr.IV were detected only in colonies (a) and (b) (Supplementary Fig. S2A). We investigated abnormalities in other chromosomes and found that, in colonies (a) and (b), a 130kb centromere-containing region of chr.VI (extending from a Ty2 at coordinate ∼140 000) was duplicated (Supplementary Fig. S2B). Furthermore, in colonies (c)–(e), chr.II was fully duplicated (Supplementary Fig. S2B).

Although the duplicated region of chr.IV was identified, the precise structure of the partial duplication and the recipient of the translocation in colonies (a) and (b) could not be determined by short-read genome sequencing. To investigate this, we performed whole genome Oxford Nanopore sequencing on colony (b) [40, 41]. Chromosome rearrangements were detected by finding reads that map to more than two distinct regions within the reference genome. To visualize these reads and to examine the generality of such rearrangement, we generated custom reference sequences and mapped the reads to them. While the two pairs of Ty2 and Ty1 in chr.IV are very similar in sequence, Ty2-2 and Ty2-3 can be distinguished by ∼1.1% of sequence difference, and ∼1.4% for Ty1-2 and Ty1-3, which enabled us to estimate which Ty transposons took place in the rearrangements and which ones overwrite the others in such events. Multiple reads from *sic1Δ* colony (b) aligned successfully to the reference predicting partial duplication of chr.IV through recombination between Ty transposons, where reads from WT colony did not align to such reference. Thus, as expected, the duplicated regions were arranged in tandem within chr.IV (Supplementary Fig. S3A). Furthermore, the duplicated region of chr.IV was translocated to the centromere-containing fragment of chr.VI, forming a fusion chromosome mediated by recombination between their Ty2 elements (Supplementary Fig. S3B). The partially duplicated region of chr.IV was also translocated to a fragment of chr.XIII via the Ty1 transposons (Supplementary Fig. S3C) on the both ends of the chr.XIII fragment, resulting in a *de facto* inversion of chr.IV sequence. As noted, in the *sic1∆* (a), (b) read coverage of the region distal to the duplicating region (extending to the telomere) also doubled. However, the increase cannot be explained solely by the translocation to chr.VI. Taken together, we hypothesized that the duplicated distal region of chr.IV, the centromere-containing region of chr.VI, and a fragment of chr.XIII all form a single new chromosome (Supplementary Fig. S3C). Based on this prediction, we designed probes for both the partially duplicated region and the distal region of chr.IV and performed Southern hybridization (Supplementary Fig. S3D). As expected, the signals labeled by the two probes were consistent and there was only one type of signal distinct from chr.IV in each colony. This indicates that the translocations involving chr.IV, chr.VI, and chr.XIII have formed a single fused chromosome, consistent with our hypothesis (Supplementary Fig. S3E).

### Partial duplication increases *SIR4* leading to rDNA instability

**Figure 3.**
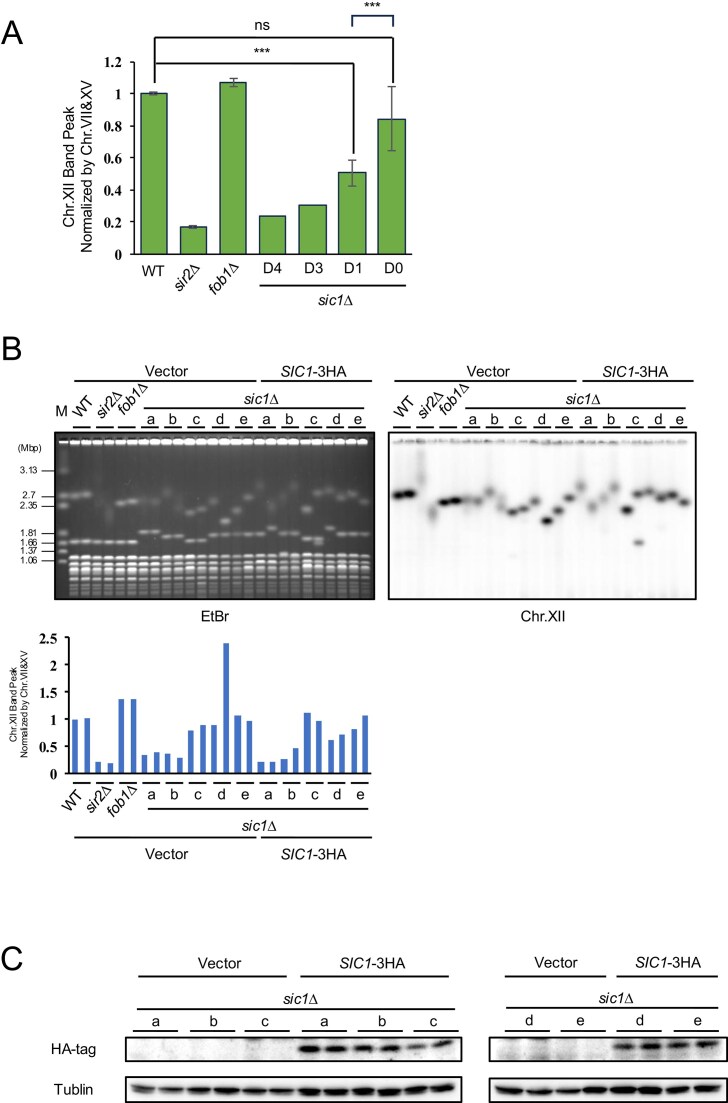
rDNA instability in *sic1Δ* is independent of *SIC1* deletion. (**A**) Quantification of the band intensity of chr.XII in Fig. 1D normalized by that of chr.VII and XV. Colonies of the *sic1∆* were classified based on their fold change of the chr IV partial duplication (Supplementary Fig. S2A). Sample size: control (WT, *sir2∆, fob1∆*); *n* = 3, D4; *n* = 1, D3; *n* = 1, D1; *n* = 7, D0; *n* = 6. Note that for D4 and D3, *n* = 1 because no additional transformations could be obtained. Error bars show the standard error of the mean (SEM) among biological replicates. Statistical significance was determined using Tukey’s honestly significant difference test following one-way ANOVA. *P* < .05 was considered statistically significant. (**B**) A single-copy plasmid with (*SIC1*-3HA) or without (vector) *SIC1* was transformed into the *sic1∆* used for WGS in Fig. 2. The genomic DNA of strains with plasmids was monitored by PFGE and Southern blotting as in Fig. 1C. The graph quantifies the band intensity of chr.XII normalized by that of chr.VII and chr.XV. (**C**) Protein level of Sic1 from the *SIC1* plasmid was confirmed by western blotting.

The stability of rDNA can be evaluated by separating chromosomes using PFGE [42]. When rDNA is unstable due to frequent copy number changes, the size of chr.XII becomes heterogenous within the cell population, causing the band to appear smeared [17]. For example, deletion of *SIR2*, which suppresses the transcription of noncoding RNA from E-pro, causes rDNA instability, resulting in a smeared chr.XII band compared to the WT (Fig. 1B and D). Conversely, deletion of *FOB1*, which induces DSBs at the RFB, stabilizes rDNA and results in a sharp chr.XII band (Fig. 1A and D). In the *sic1∆* analysis (Fig. 1D), the mutant exhibited both smeared (lanes 11, 12, 17–19, 23–25) and sharp (lanes 13–16, 20–22) chr.XII bands. Notably, colonies containing partial duplications of chr.IV always exhibited smeared chr.XII bands. In particular, the bands for colonies (a) and (b) were smeared to a degree comparable to that of the *sir2∆*, suggesting a correlation between partial duplication of chr.IV and rDNA instability. To investigate this, colonies were grouped based on the number of chr.IV partial duplications, and the intensity of chr.XII band peaks in Fig. 1D was quantified. Lower peak intensity indicates a more diffused (smeared) band, while higher intensity indicates a sharper band [43]. A negative correlation was observed between the duplication number (D4–D0) and the band intensity (Fig. 3A). This suggests that rDNA instability is a secondary phenotype of the genomic rearrangement rather than a direct effect of the *SIC1* deletion.

To confirm this, we introduced the *SIC1*-3HA plasmid (expressed under its native promoter and terminator) into the colonies used for WGS [44]. Despite “the reintroduction of* SIC1*,” the chr.XII band remained smeared in colonies (a), (b), (d), and (e) (Fig. 3B and C), confirming that rDNA instability is not a direct consequence of *SIC1* absence.

**Figure 4.**
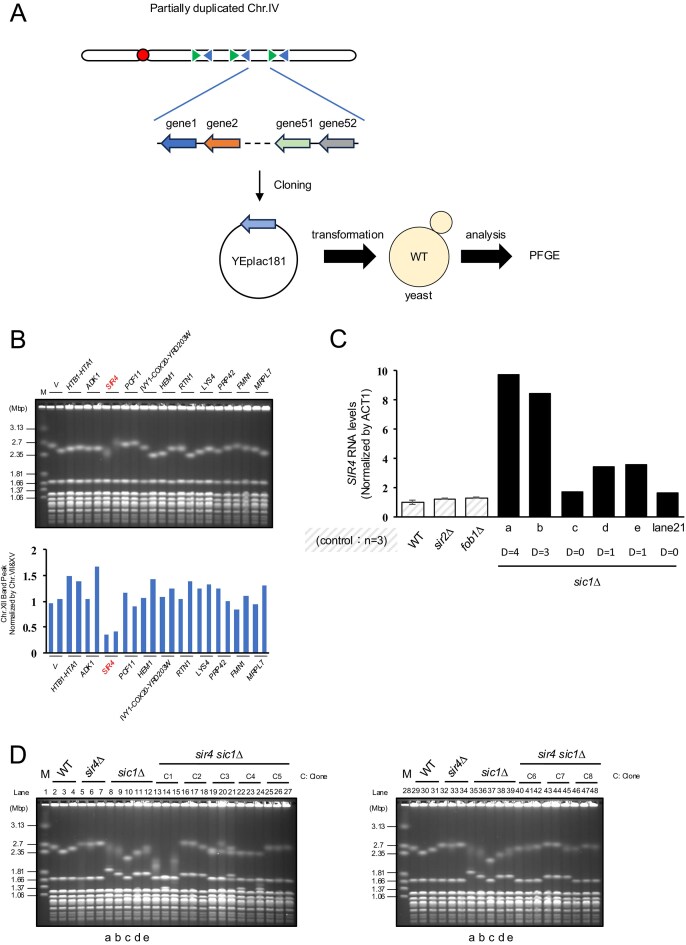
rDNA instability is caused by increased number of *SIR4* genes. (**A**) Identification of the causative gene for the rDNA instability. Fifty-two genes in the duplicated region were cloned into multicopy plasmids and transformed into WT strains, respectively. Their genomic DNA was separated by PFGE, and analyzed whether these genes induce rDNA instability. (**B**) PFGE analysis of plasmid-transformed strains. Chr.XII appeared smeared (indicated by low intensity of chr XII band) in strains transformed with *SIR4* plasmid. The graph quantifies the band intensity of chr.XII normalized by that of chr.VII and chr.XV. (**C**) The mRNA level of *SIR4* was analyzed by RT-qPCR for the strains in Fig. 2. As a control, a colony without duplication (Fig. 1C, lane 21) was used. Note that for D4 and D3, *n* = 1 because no additional transformations could be obtained. (**D**) PFGE analysis of the *sic1∆* in *sir4∆* background, three colonies were picked for each of the eight transformants. Colonies for *sic1Δ* were those used in Fig. 2. Six colonies derived from two independent clones exhibited Chr IV elongation, but not Chr XII instability (lanes 16–18 in both panels).

These results imply that rDNA instability in the *sic1∆* is driven by the increased dosage of gene(s) located within the duplicated region of chr.IV. To identify the causative gene(s), we cloned all 52 genes in the duplicated region into multicopy plasmids. Plasmids containing 1–3 genes each were transformed into WT cells and their genomes were separated by PFGE (Fig. 4A). rDNA instability was induced specifically in cells carrying *SIR4*-containing plasmids (Fig. 4B and Supplementary Fig. S4A). One transformant carrying a *BTT1*-containing plasmid showed a weaker chr.XII signal. However, this was due to band splitting, a phenotype distinct from the smearing induced by *SIR4* overexpression. We further confirmed via reverse transcription quantitative PCR (RT-qPCR) that *SIR4* mRNA expression levels correlated with the copy number of the chr.IV duplication (Fig. 4C). A colony without duplication (Fig. 1C, lane 21) was used as a control.

Finally, we examined whether *SIR4* deletion restores rDNA stability in *sic1∆* mutants harboring chr.IV partial duplications. We analyzed 24 colonies from 8 clones of a *sir4∆ sic1∆* double mutant (Fig. 4D). Partial duplication of chr.IV was observed in clone 2 (left, lanes 16–18) and clone 7 (right, lanes 16–18) (Fig. 4D). These colonies exhibited sharper chr.XII bands compared to the single *sic1∆* colonies (d) and (e) (Supplementary Fig. S4B). These results further confirm that rDNA instability in the *sic1∆* with duplication of chr.IV is caused by the overexpression of *SIR4*. Although some *sir4∆ sic1∆* colonies displayed smeared bands in the absence of chr.IV duplication, this phenotype likely resulted from a stochastic reduction in rDNA copy number, which activates noncoding RNA transcription from E-pro (Fig. 4D left, lanes 13 and 15). [3]

Mechanistically, Sir4 recruits Sir2 to telomeres and *MAT* loci [30–32], while the regulator of nucleolar silencing and telophase exit (RENT) complex (containing Sir2, Cdc14, and Net1) silences the rDNA IGS region [45]. Previous studies indicate that *SIR4* expression levels regulate Sir2 localization [46]. Therefore, we propose that overproduction of Sir4, driven by chr.IV partial duplication, titrates Sir2 away from the nucleolus, disrupting IGS silencing and leading to rDNA instability.

### *SIR4* overexpression upregulates E-pro transcription

**Figure 5.**
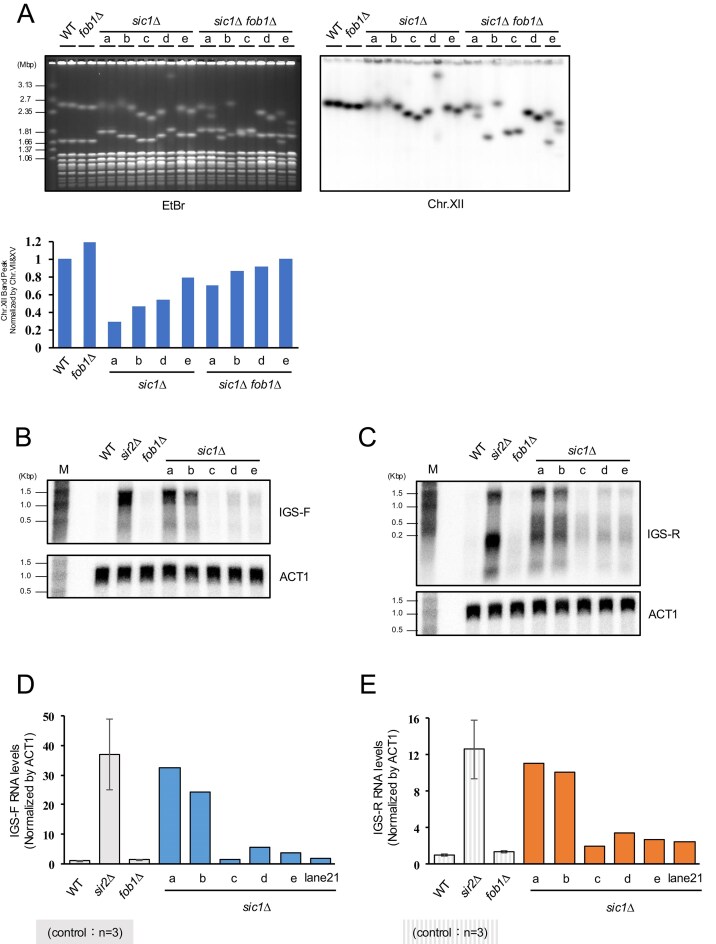
Overexpressed *SIR4* increases the E-pro transcription. (**A**) *FOB1* was deleted from the *sic1∆* colonies used for WGS in Fig. 2, and their genomic DNA was separated by PFGE. The graph displays the average chr.XII band intensity (*n* = 2 colonies) normalized by that of chr.VII and chr.XV. Colony (c) was excluded from quantification due to the overlap of chr.XII and chr.IV bands. In cases where chr.XII appeared as split bands, the intensities of both bands were summed. The amount of noncoding RNA from IGS was evaluated by northern blotting (**B** and **C**) and RT-qPCR (**D** and **E**). Note for D4 and D3, *n* = 1 because no additional transformations could be obtained.

Before assessing E-pro expression, we investigated whether rDNA instability in *sic1∆* is dependent on the Fob1-mediated RFB. Fob1 binding at the RFB is the initiating event for rDNA instability, leading to the formation of DSBs [7–11]. To determine whether rDNA instability in the *sic1∆* occurs in a Fob1-dependent manner, we deleted *FOB1* from the colonies used for WGS. PFGE analysis revealed that *sic1∆ fob1∆* double mutants exhibited sharper chr.XII bands compared to the smeared bands of *sic1∆* single mutants (Fig. 5A). In some colonies of the *sic1∆ fob1∆*, the chr.XII band appeared as two distinct bands. This could result from either a single cell harboring two chr.XII copies or a heterogeneous population comprising two distinct cell types with different rDNA lengths. Southern blotting showed that colonies with two chr.XII bands had weaker signals for each split band compared to colonies with a single band (Fig. 5A). This suggests the presence of a mixed population where each subpopulation maintains a distinct, stable rDNA length. We therefore concluded that rDNA instability in *sic1∆* is dependent on Fob1.

Next, we examined transcription from the bidirectional promoter, E-pro, which drives rDNA instability downstream of Fob1-dependent DSB formation (Fig. 1B) [12]. E-pro is located ∼260 bp from the RFB and generates noncoding transcripts (IGS-F and IGS-R); these transcripts inhibit cohesin association, thereby promoting unequal sister-chromatid recombination and rDNA instability (Fig. 1B). Northern blotting revealed that the E-pro expression was increased in the *sic1∆* with partial duplication of chr.IV. Moreover, the expression levels appeared to increase in proportion to the number of partial duplications (Fig. 5B and C). To validate this more quantitatively, we measured the E-pro expression level using RT-qPCR. The results confirmed a strong positive correlation between the number of chr.IV partial duplications and E-pro expression levels (Fig. 5D and E). To verify that this effect was driven by *SIR4* gene dosage, we analyzed strains transformed with *SIR4* plasmids (Supplementary Fig. S5). As expected, *SIR4* expression levels correlated directly with E-pro transcript abundance. Collectively, these results suggest that partial duplication of chr.IV leads to *SIR4* overexpression, which triggers E-pro transcription and subsequent rDNA instability.

### Ty2–Ty1 inverted repeat forms a hairpin structure, leading to DSBs.

While rDNA instability in *sic1∆* appears to be a secondary effect, the partial duplication of chr.IV seems to be a direct consequence of *SIC1* deletion. This duplication occurs between inverted Ty2 and Ty1 repeats (Fig. 2B). Ty elements are repetitive sequences that are dispersed throughout the genome [21]. They are known as hotspots for ectopic recombination, leading to chromosomal rearrangements [47]. Our sequencing data support the hypothesis that ectopic recombination, mediated by homology between these Ty2–Ty1 inverted repeats, drives the partial duplication (Fig. 2 and Supplementary Fig. S3 and S6A).

When recombination events occur, they are typically triggered by the formation of DSBs. Nanopore sequencing indicated that the duplicated region of chr.IV was translocated to chr.VI via the distal Ty2 element (Ty2-3) (Fig. 1A and Supplementary Fig. S3B). Ty1 and Ty2 show high sequence similarity, particularly within the 334 bp LTR located at either ends [21]. The opposing LTRs of Ty2-3 and Ty1-3 exhibit ∼95% sequence identity and are separated by a spacer of only 21 bp (Fig. 1C and Supplementary Fig. S6B). This configuration is likely to form a hairpin structure, creating a fragile site prone to DSBs often caused by replication-associated topological stress. To investigate DSB formation at this locus, we digested genomic DNA with *StuI* and *NcoI* and analyzed the 17 579 bp region using specific probes (Supplementary Fig. S6B).

*Sic1∆* colonies without chr.IV duplication were used to ensure that the baseline chromosomal structure matched the WT. If DSBs occur in the middle of the hairpin, fragmented bands of 10 265 and 7314 bp are expected. Indeed, bands corresponding to these DSB fragments (red arrows: ∼10 kbp and ∼7 kbp) were observed in *sic1∆* (Supplementary Fig. S6B). Additionally, a ∼14 kb band (indicated by an asterisk) was observed exclusively in the *sic1∆*, likely reflecting the subpopulation of cells that had already undergone partial duplication. Unexpectedly, bands of DSB fragments (red arrows) were also detected in the WT with intensity comparable to those of the *sic1∆*. This finding indicates that while DSBs occurs at the hairpin structure formed by Ty2-3 and Ty1-3, they are properly repaired in the WT. The high frequency of rearrangements in *sic1∆* suggests that Sic1 plays a crucial role for the error-free repair of DSBs at this complex locus.

### DSBs occur in a replication-dependent manner, while ectopic recombination is induced by transcription

We hypothesized that the formation of hairpin structures leads to replication stress and topological stress, inducing DSBs (Supplementary Fig. S7A). To examine this, we first tested the replication dependency by comparing the DSB signals in the exponential (log) and stationary phases. We observed that the putative DSB signal decreased in the stationary phase compared to the exponential phase (Supplementary Fig. S7B). This suggests that the DSBs are replication-dependent and likely result from fork stalling at the hairpin structure. Next, we investigated whether Ty transcription induces topological stress, which leads to DSBs (Supplementary Fig. S7A). To test this, we deleted *SIC1* in the *SPT3* deletion background. *SPT3* is a transcription factor that activates Ty transcription [48]. We selected colonies without chr.IV partial duplication from the *spt3∆ sic1∆* double mutant and performed the DSBs assay. The double mutant showed no change in the DSB signal intensity compared to the *sic1∆* single mutant (Supplementary Fig. S7B), indicating that Ty transcription is independent from the formation of DSBs.

In the rDNA locus, E-pro transcription does not increase DSBs but stimulates ectopic recombination [12]. Therefore, we hypothesized that Ty transcription might similarly promote ectopic recombination to drive chr.IV partial duplication. First, we evaluated Ty1 and Ty2 transcript levels in the *sic1∆* colonies used for WGS via RT-qPCR. Ty1 and Ty2 expression tended to be higher in the *sic1∆* compared to that of the WT (Supplementary Fig. S7C). In particular, Ty2 expression levels were also increased in the *sic1∆* colony (c) and lane21, which do not show a partial duplication of chr. IV. This suggests that the increased Ty2 expression in the *sic1∆* is a direct consequence of *SIC1* deletion, rather than an increase in the Ty2 genome copy number by duplication of chr. IV. [49]

Finally, we assessed whether this Ty transcription promotes the actual recombination event. We evaluated the frequency of chr.IV partial duplication in *spt3Δ sic1Δ* double mutants by PFGE. We analyzed 15 colonies (3 colonies from 5 independent transformants) across 3 independent *spt3Δ* clones, totaling 45 colonies (Supplementary Fig. S7D). Only one clone showed partial duplication of chr.IV (Supplementary Fig. S7D, top right). This reduction of duplication event suggests that Ty transcription promote ectopic recombination, possibly by mechanisms such as cohesin dissociation.

## Discussion

In this study, we aimed to elucidate the relationship between the stability of the rDNA locus and other genomic regions. To this end, we reanalyzed the YSRD and identified the *sic1∆* mutant, which exhibits both rDNA instability and chr.IV elongation (Fig. 1D) [18–20]. We revealed that the elongation of chr.IV in the *sic1∆* strain is due to a partial duplication caused by ectopic recombination mediated by Ty2–Ty1 inverted repeats (Fig. 2A). Our analysis clarified the distinct mechanisms underlying these instabilities: the partial duplication of chr.IV triggers a secondary effect where increased *SIR4* gene dosage leads to overexpression of Sir4. This overexpression upregulates E-pro transcription, thereby inducing recombination and instability in the rDNA. On the other hand, the chr.IV rearrangement itself is initiated by DSBs at the Ty2–Ty1 inverted repeats, followed by translocation.

We determined that rDNA instability in the *sic1∆* is a secondary effect of the partial duplication of chr.IV. The duplicated region contains the *SIR4* gene, leading to increased expression levels (Fig. 4C). Sir4 forms a complex with Sir2, which is involved in silencing telomeres and the MAT loci [30–32]. Therefore, we speculate that excess Sir4 titrates Sir2 away from the nucleolus, depleting the RENT complex. This loss of Sir2 at the rDNA allows for E-pro transcription, resulting in instability. Previous studies have reported that nearly half of the mutants with chromosomal abnormalities other than rDNA also exhibit rDNA instability [19].Our data suggest that for many of these strains, rDNA instability may be a secondary side effect of aneuploidy or rearrangements rather than a direct phenotype.

While we clarified the effect of chr.IV partial duplication on rDNA stability, it is also possible that rDNA status influences chr.IV stability. Colonies with partial duplication of chr.IV exhibited higher rDNA copy numbers compared to those without duplication (Fig. 1D). High rDNA copy number, which also requires high amounts of proteins for replication and repair, would titrate genome stabilizing factors for other genomic regions. Although previous studies have suggested that high copy number rDNA increases resistance to mutagens [50], under conditions of Sic1 depletion, maintaining a high copy number may no longer be advantageous and could contribute to global genomic stress.

Previous studies have also suggested that Sic1 contributes to genome stability by ensuring sufficient time in G1 phase for pre-RC formation [29]. Consequently, replication initiation efficiency would be affected by the *sic1* mutation. Replication origins of budding yeast are distributed at intervals of 40–100 kb [51–53]. However, within the duplicated region, three replication origins are arranged at unusually close intervals (∼10 kb). In contrast, the distance from these replication origins to the nearest external replication origin is ∼90 kb. Genomic regions with sparse replication origins are known to be fragile and prone to rearrangement [54]. Furthermore, the region surrounding the duplication is rich not only in Ty elements but also in tRNA genes (tDNAs, Fig. 1C) [55]. Highly transcribed tDNAs are known to stall replication forks [56–58]. We suppose that the characteristic landscape of this locus creates a hotspot for replication stress, leading to the observed partial duplication.

The investigation of the DSBs responsible for the partial duplication revealed that they occur at the center of the Ty2–Ty1 inverted repeat (Supplementary Fig. S6B). Due to the high sequence similarity between the LTRs of Ty2 and Ty1, these regions can form hairpin structures (Supplementary Fig. S6B). Such hairpin structures induce topological stress or stall replication fork, leading to DSBs. Our results indicate that replication stress is the primary driver of these DSBs. However, these DSBs were observed in both *sic1∆* and WT strains (Supplementary Fig. S6B), suggesting that DSBs occur at a certain frequency but are properly repaired in the WT, likely through homologous recombination with the sister chromatid. Sic1 appears to play a critical role in this repair mechanism, which is why the *sic1∆* exhibit partial duplication of chr.IV. Ectopic recombination is generally repressed by the cohesion and catenation of sister chromatids. In the rDNA, E-pro transcription releases cohesin and stimulates ectopic recombination [12] (Fig. 1B, right). In the *sic1∆*, Ty2 transcription was particularly increased (Supplementary Fig. S7C). Moreover, previous studies have shown that cohesin binds to Ty2. [59] These suggest that the increased frequency of ectopic recombination in the *sic1∆* after DSB formation at Ty2 may be due to cohesin dissociation caused by the upregulated Ty2 transcription.

Cohesion is regulated throughout the cell cycle. In budding yeast, cohesin consists of Smc1–Smc3 heterodimers bound to each sister chromatid, linked by Mcd1 and Irr1 [60–63]. Mcd1 accumulates in cells during late G1 phase, and cohesin is loaded onto chromatin by Scc2–Scc4 during the same phase [60, 64]. During S-phase, Eco1-mediated acetylation of Smc3 stabilizes the complex, tethering sister chromatids until anaphase onset, where Mcd1 is cleaved by separase (Esp1), leading to chromosome segregation at anaphase [62, 65]. Additionally, Smc3 deacetylation by Hos1 enables the reuse of Smc1–Smc3 in the next cell cycle [66–68]. Since these processes are tightly regulated by cyclin-CDK, Sic1—a CDK inhibitor—likely plays a significant regulatory role. On the other hand, although rDNA stability is highly dependent on cohesin [12], rDNA instability in the *sic1∆* was not directly caused by *SIC1* deletion. This could be explained by competition for cohesin resources. If cohesin preferentially binds to the rDNA, high rDNA copy number would titrate cohesin and lead to depletion of cohesin for other genomic regions. This would be consistent with the chr IV-elongated strains with high rDNA copy numbers. Finally, catenation of DNA, which helps to tether sister chromatids, results from the collision of replication forks [69]. The three replication origins located within the duplicated region of chr.IV may facilitate the formation of catenated DNA, tethering sister chromatids, in the WT. However, the frequency of replication origin firing is suggested to be reduced in the *sic1∆*. This could prevent the formation of catenated DNA, thereby weakening sister chromatid tethering and further promoting ectopic recombination.

## Supplementary Material

gkag096_Supplemental_File

## Data Availability

The data, additional information, and materials will be shared on reasonable request to the corresponding author. Data have been uploaded to https://doi.org/10.17632/dds7tp65bd.1.
