# Supplementary material for: sic1 mutation leads to rDNA instability by partial duplication with SIR4
Source: Nucleic Acids Res. 2026 Feb 10;54(4):gkag096. doi: 10.1093/nar/gkag096 (PMC12887534; doi:10.1093/nar/gkag096)
Supplement: gkag096_Supplemental_File [file gkag096_supplemental_file.pdf]

SUPPLEMENTARY DATA

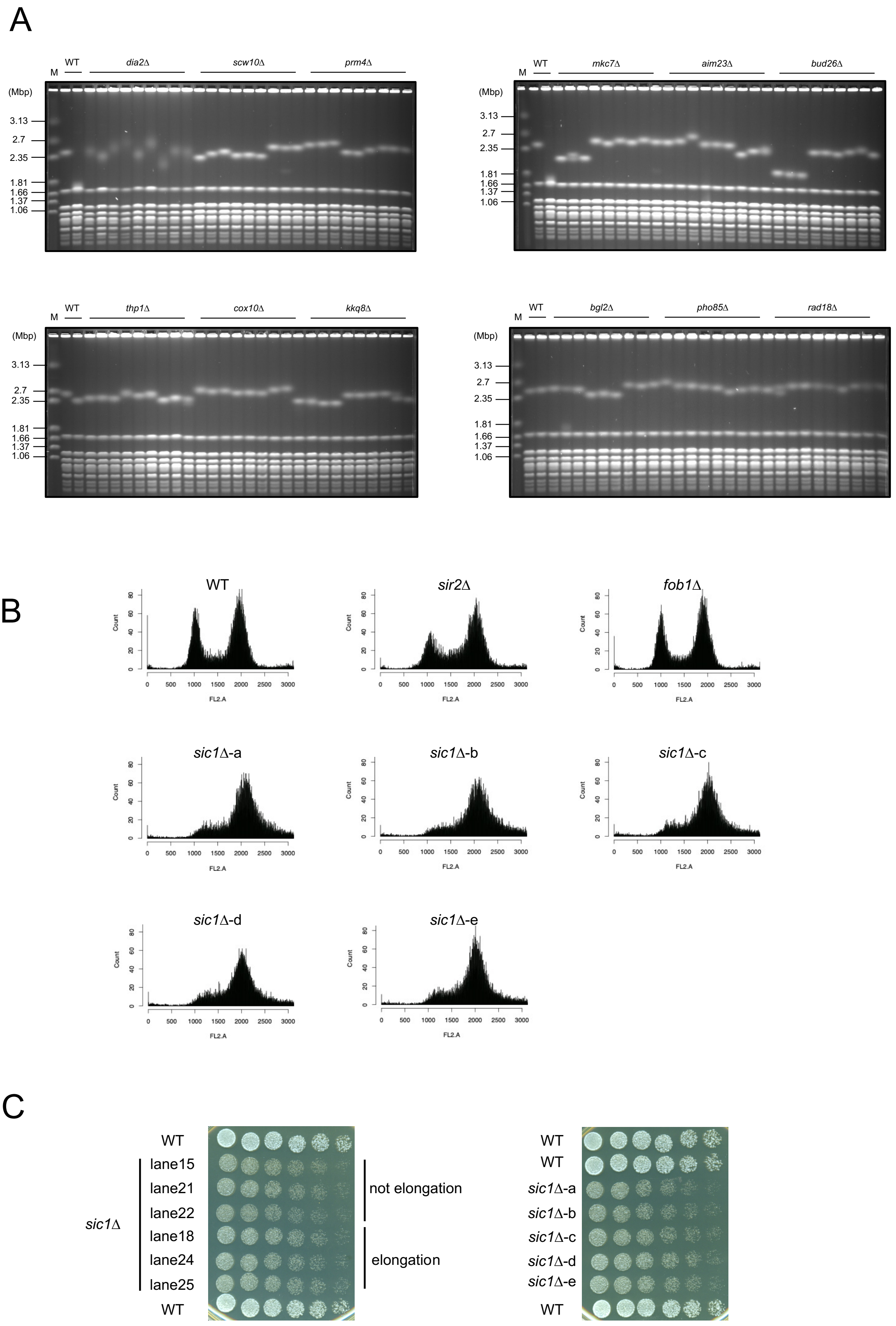

Supplementary Figure 1: Confirmation of the reproducibility of chr.IV elongating mutants. (A) Genomic DNA of candidate mutants were separated by PFGE and observed by ethidium bromide. (B) The plots of flowcytometry analysis for *sic1*Δ. (C) Serial dilution growth assay in *sic1*Δ. Colonies from Figure 1D were used.

A

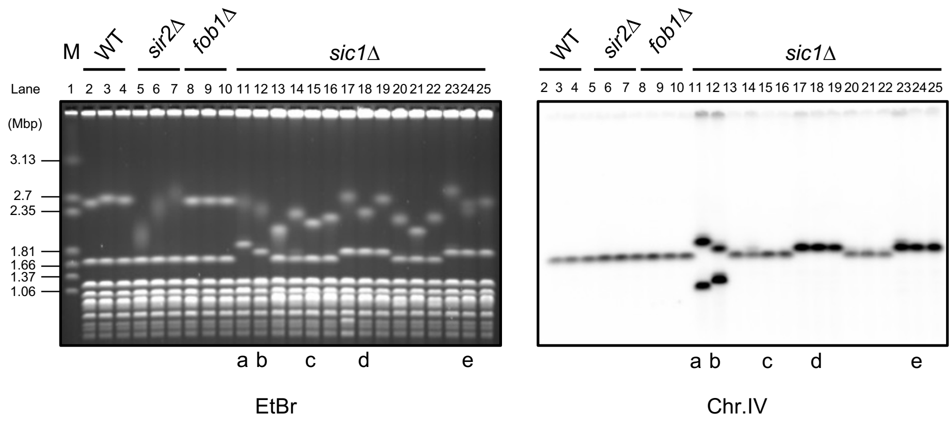

B

***sic1*Δ**	**a**	**b**	**c**	**d**	**e**
**Chr.II**	**-**	**-**	**full**	**full**	**full**
**Chr.VI**	**partial**	**partial**	**-**	**-**	**-**

Supplementary Figure 2: Chromosome abnormalities observed in *sic1Δ*. (A) Southern blotting was performed using the chr.IV probe in Figure 1D. (B) Duplicated regions found in WGS.

A

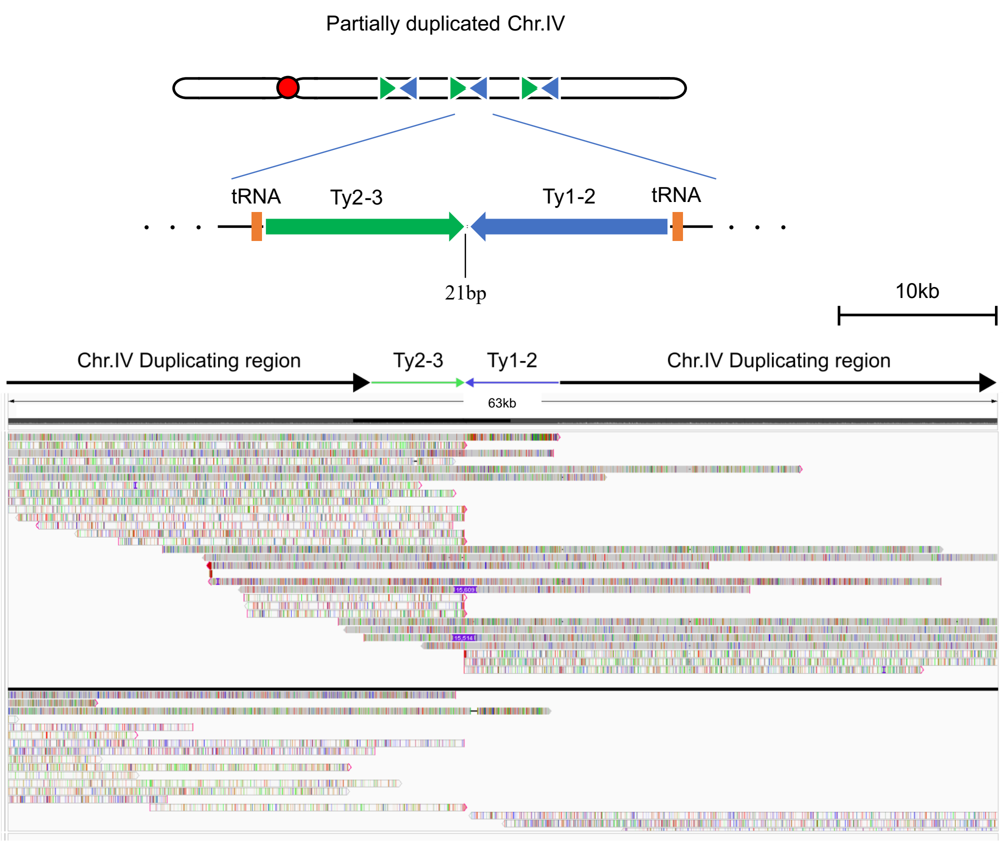
Partially duplicated Chr.IV
tRNA
Ty2-3
Ty1-2
tRNA
21bp
10kb
Chr.IV Duplicating region
Ty2-3
Ty1-2
Chr.IV Duplicating region
63kb
sic1Δ b
WT

B

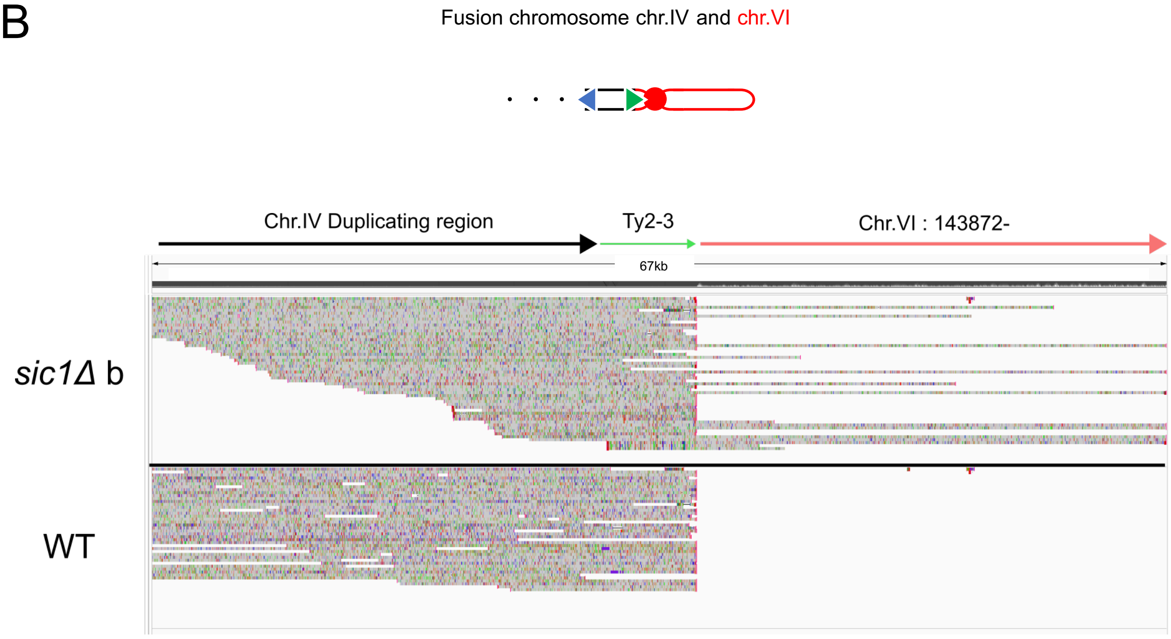
Fusion chromosome chr.IV and chr.VI
Chr.IV Duplicating region
Ty2-3
Chr.VI : 143872-
67kb
sic1Δ b
WT

C

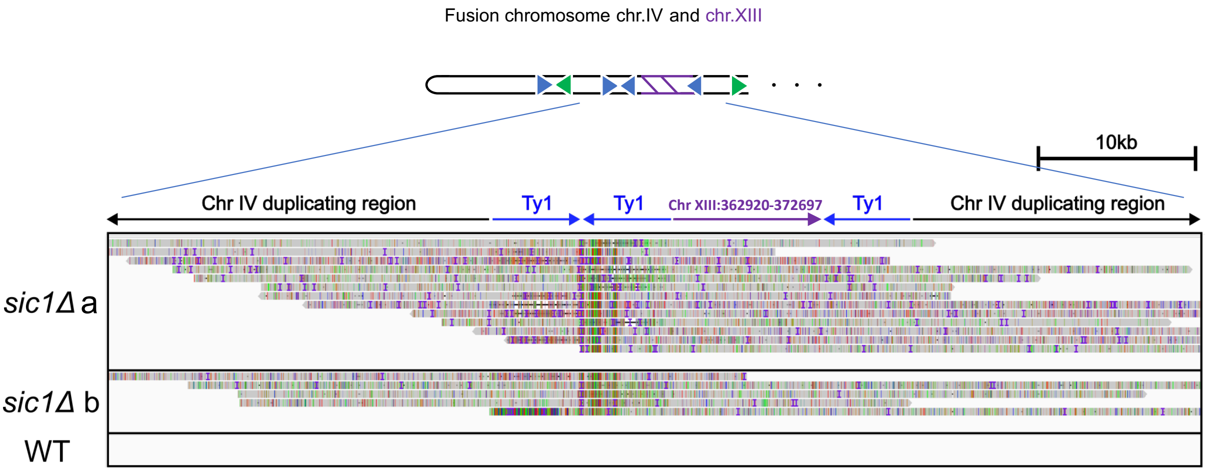

D

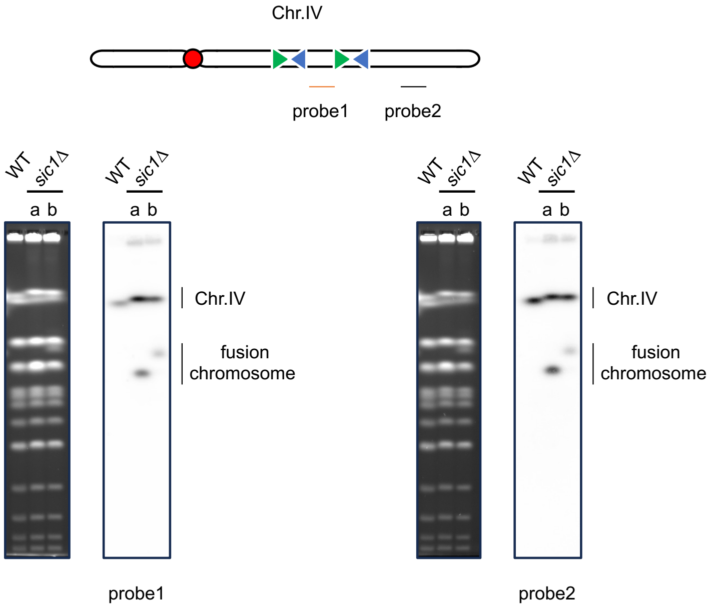

E

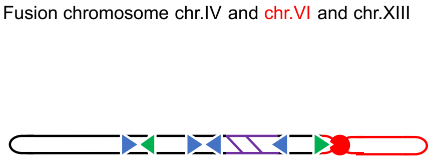

Supplementary Figure 3: Analysis of chr.IV rearrangement with Oxford Nanopore sequencer. (A) Sequenced reads were aligned to a reference sequence representing the tandem duplication, specifically spanning the junction between the Ty2-3 and Ty1-2 elements. Individual mapped reads spanning the boundary between the two duplicated segments are displayed. (B) Reads were also aligned

to a reference genome representing the translocation between chr.IV and chr.VI, mediated by the shared Ty2 element. (C) The distal segment of Chr.IV, including the duplicated region, was inverted and translocated to Chr.XIII via Ty1-mediated recombination on the both sides. (D) Southern hybridization results were obtained by using probes for the duplicating region of chr. IV and the region distal to that region. PFGE was performed using switching parameters optimized to resolve the fusion chromosome, distinct from the conditions used for rDNA separation. (E) Schematic of the predicted *sic1*Δ-b fusion chromosome structure based on the combined results of Nanopore sequencing and Southern hybridization results.

A

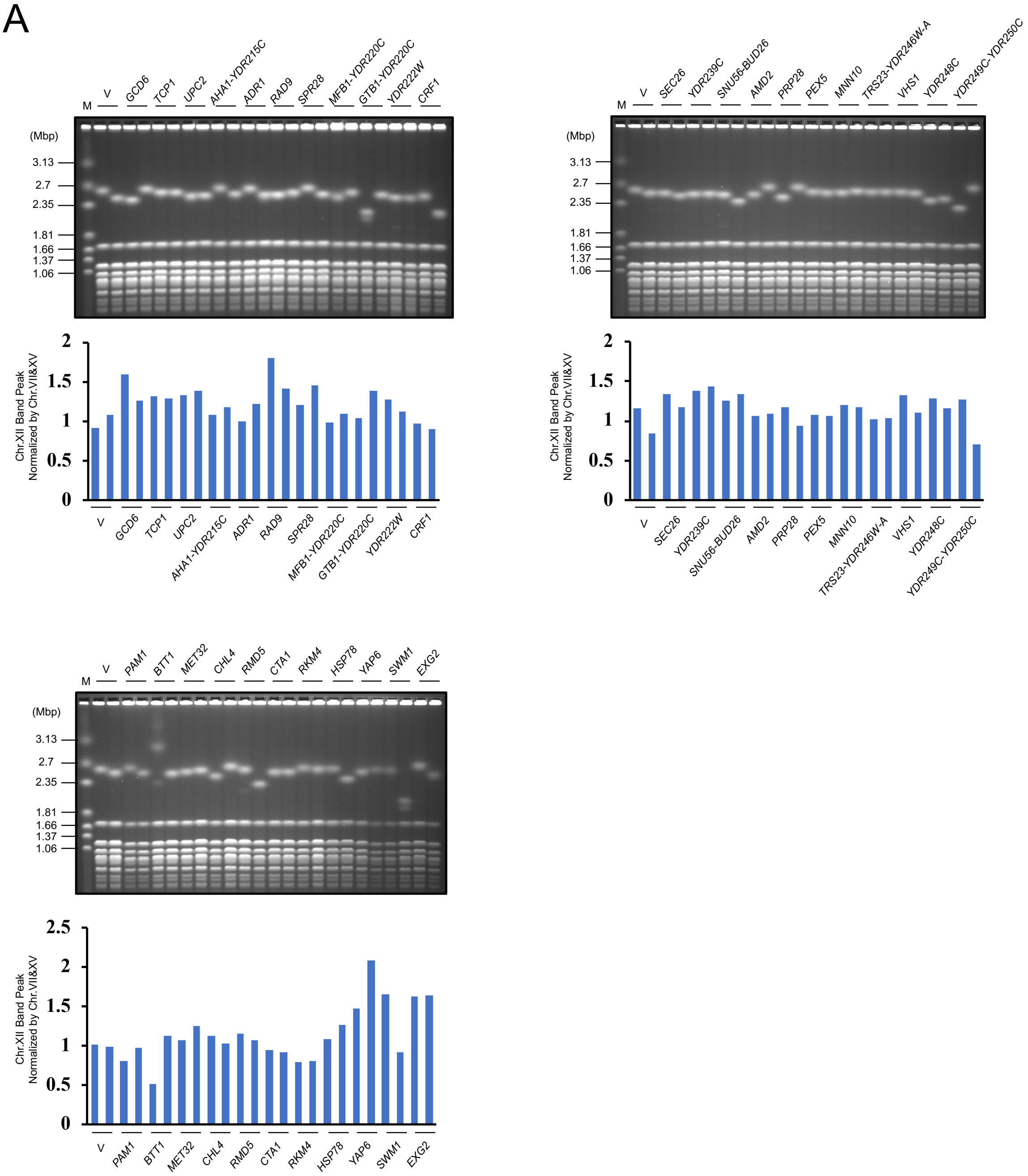
M
V
GCD6
TCP1
UPC2
AHA1-YDR215C
ADR1
RAD9
SPR28
MFB1-YDR220C
GTB1-YDR220C
YDR222W
CRF1
(Mbp)
3.13
2.7
2.35
1.81
1.66
1.37
1.06
Chr.XII Band Peak
Normalized by Chr.VII&XV
SEC26
YDR239C
SNU56-BUD26
AMD2
PRP28
PEX5
MNN10
TRS23-YDR246W-A
VHS1
YDR248C
YDR249C-YDR250C
PAM1
BTT1
MET32
CHL4
RMD5
CTA1
RKM4
HSP78
YAP6
SWM1
EXG2

B

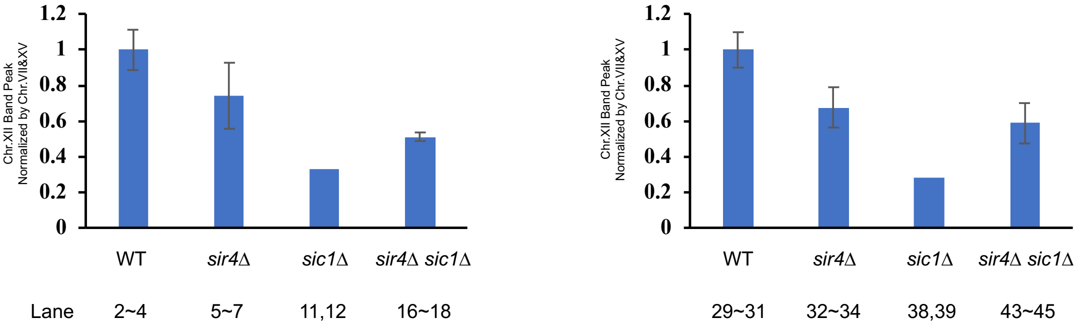
Chr.XII Band Peak
Normalized by Chr.VII&XV
WT
sir4Δ
sic1Δ
sir4Δ sic1Δ
Lane
2~4
5~7
11,12
16~18
29~31
32~34
38,39
43~45

Supplementary Figure 4: Identification of the causative gene in the duplicated region of chr.IV for rDNA instability. (A)PFGE analysis for the plasmid transformed strains other than shown in Figure 4B. The quantitative graph shows the band intensity of chr.XII normalized by that of chr.VII and chr.XV. (B) Quantitative graph in Figure 4D. The quantitative graph shows the band intensity of chr.XII normalized by that of chr.VII and chr.XV. The quantified *sic1*Δ and *sir4*Δ *sic1*Δ colonies showed a partial duplication (D=1) of chr.IV. The quantified lane is listed below.

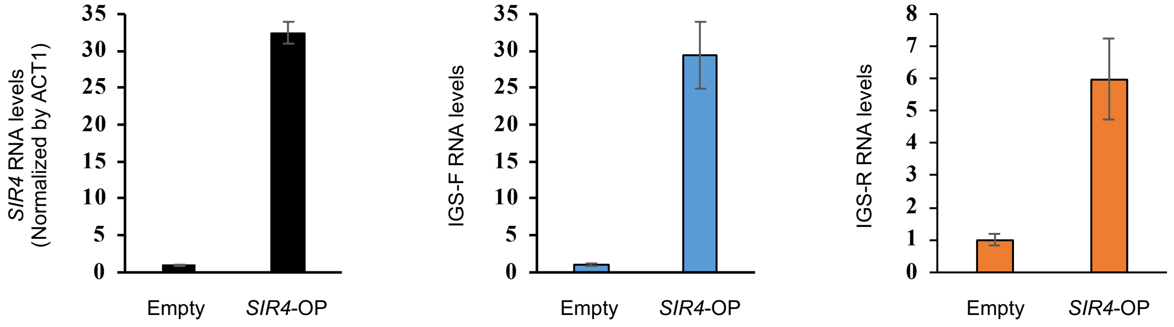

Supplementary Figure 5: The E-pro transcription is increased in *SIR4*-overexpressed cells. RNA level in cells with *SIR4* overexpression plasmid was analyzed by RT-qPCR. Left: *SIR4*, middle: IGS-F, right: IGS-R. Error bars indicate SEM among 3 biological replicates.

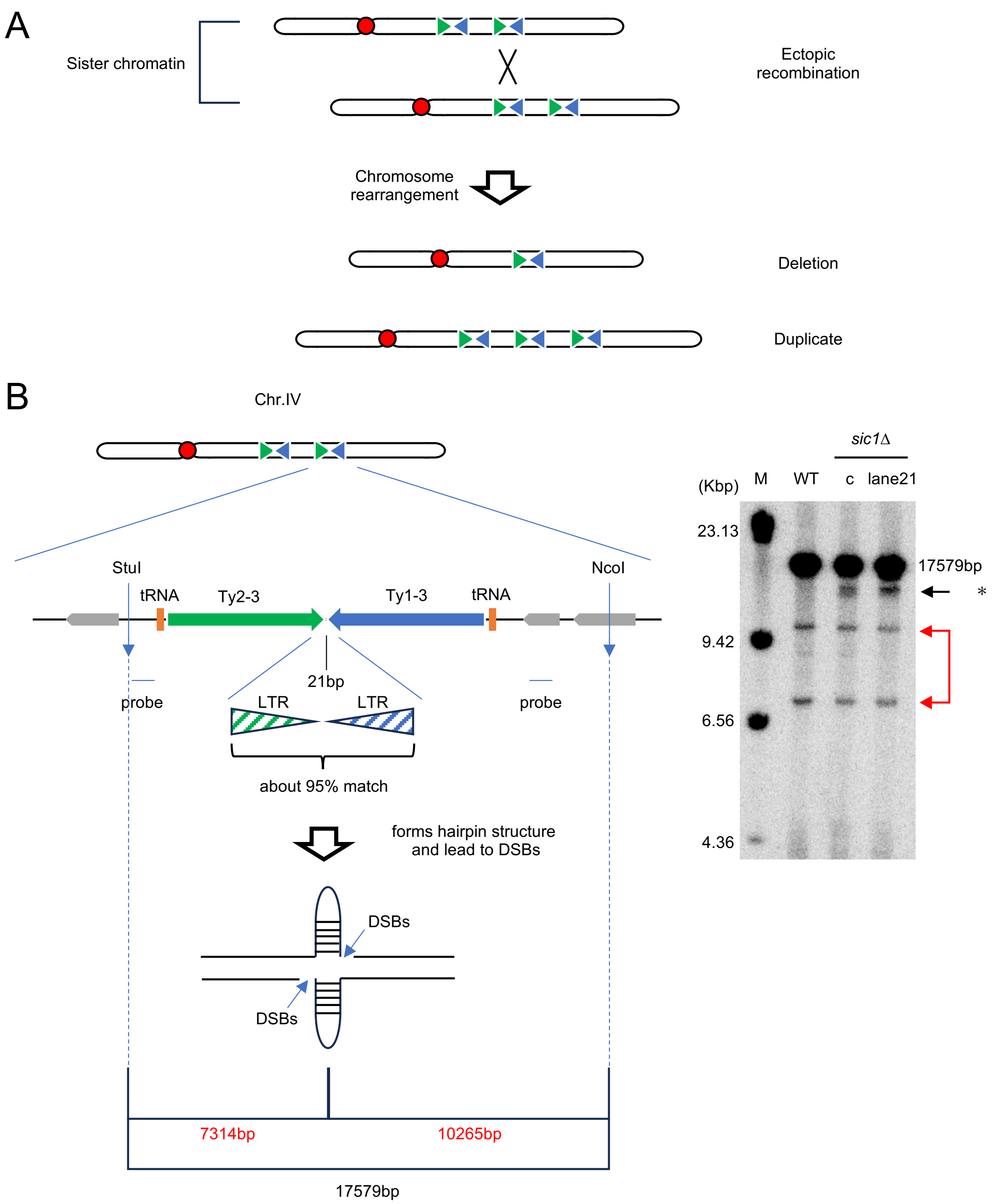

Supplementary Figure 6: Ty2-3 and Ty1-3 inverted repeat forms a hairpin structure that leads to DSBs. (A) A model for the mechanism of chr.IV partial duplication. It was suggested that chromosomal rearrangements occurred through ectopic recombination mediated by Ty2-Ty1 inverted repeats. (B) Left: The Inverted repeat which consists of Ty2-3 and Ty1-3 forms a hairpin structure and leads to DSBs. Right: Genomic DNA of WT and *sic1Δ* digested with StuI and NcoI was separated by electrophoresis, and signals were detected using two probes specific to the region around Ty2-3 and

Ty1-3. Red arrow: Bands that are thought to be fragmented by DSBs due to the formation of hairpin structures. This likely arises from a partial duplication of chr.IV in some cells in *sic1*Δ.

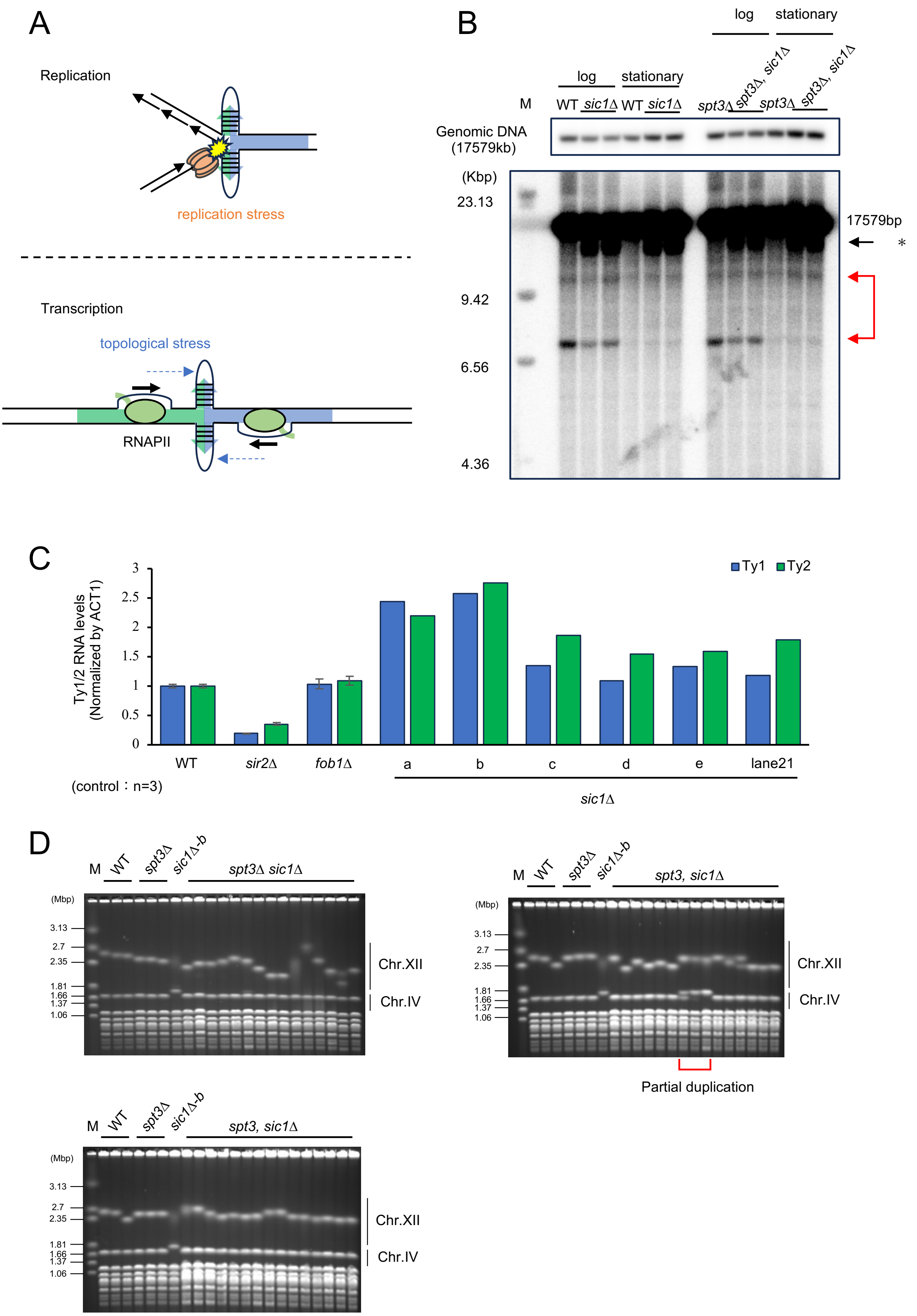

Supplementary Figure 7: DSBs occur in a replication-dependent manner and ectopic recombination is induced by Ty transcription. (A) Hypothesis on the mechanism by which hairpin structure formation leads to DSBs. (Top) We predict the occurrence of replication stress due to the collision of the replication fork with a hairpin structure, and (bottom) the occurrence of topological stress due to the progression of the transcription machinery. (B) DSBs assay to test the hypothesis of Supplementary Figure 7A. The probe used is the same as that shown in Supplementary Figure 6B. In the log phase, many cells are dividing, and in the stationary phase, most cells are in the G1 phase and no DNA replication occurs. *SPT3* is a Ty transcription factor, and its deletion results in almost no transcription. (C) The amount of Ty1 and Ty2 RNA was evaluated by RT-qPCR. (D) PFGE analysis of *sic1*Δ in *spt3*Δ background, 3 colonies were picked for each 15 transformants.

Table S1: Yeast strains

genotype	source
S. cerevisiae, BY4741 strain (wild type): YTM33, *MATa his3D1 leu2D0 met15D0 ura3D0*	Brachmann CB et al., 1998
S. cerevisiae, BY4741 strain: YTM125, *MATa his3D1 leu2D0 met15D0 ura3D0 sir2Δ::URA3*	This paper
S. cerevisiae, BY4741 strain: YTM224, *MATa his3D1 leu2D0 met15D0 ura3D0 fob1Δ::NatMX*	T Murai et al., 2024
S. cerevisiae, BY4741 strain: YTM258-262, *MATa his3D1 leu2D0 met15D0 ura3D0 sic1Δ::URA3* Clone1-5	This paper
WT colonies used in Figure 1D: YTM405-407	This study
*sir2*Δ colonies used in Figure 1D: YTM408-409	This study
*fob1*Δ colonies used in Figure 1D: YTM410-412	This study
*sic1*Δ colonies used in Figure 1D: YTM414-428	
*sic1*Δ colony (a): YTM414	This study
*sic1*Δ colony (b): YTM415	This study
*sic1*Δ colony (c): YTM418	This study
*sic1*Δ colony (d): YTM421	This study
*sic1*Δ colony (e): YTM427	This study
*sic1*Δ lane21: YTM424	This study
S. cerevisiae, BY4741 strain: YTM340-342, *MATa his3D1 leu2D0 met15D0 ura3D0 dia2Δ::URA3*	This paper
S. cerevisiae, BY4741 strain: YTM1038-1040, *MATa his3D1 leu2D0 met15D0 ura3D0 scw10Δ::URA3*	This paper
S. cerevisiae, BY4741 strain: YTM1043-1045, *MATa his3D1 leu2D0 met15D0 ura3D0 prm4Δ::URA3*	This paper
S. cerevisiae, BY4741 strain: YTM1048-1050, *MATa his3D1 leu2D0 met15D0 ura3D0 mkc7Δ::URA3*	This paper
S. cerevisiae, BY4741 strain: YTM1053-1055, *MATa his3D1 leu2D0 met15D0 ura3D0 aim23Δ::URA3*	This paper
S. cerevisiae, BY4741 strain: YTM1058-1060, *MATa his3D1 leu2D0 met15D0 ura3D0 bud26Δ::URA3*	This paper

S. cerevisiae, BY4741 strain: YTM1008-1010,<br><br>*MATa his3D1 leu2D0 met15D0 ura3D0 thp1Δ::URA3*	This paper
S. cerevisiae, BY4741 strain: YTM1013-1015,<br><br>*MATa his3D1 leu2D0 met15D0 ura3D0 cox10Δ::URA3*	This paper
S. cerevisiae, BY4741 strain: YTM1018-1020,<br><br>*MATa his3D1 leu2D0 met15D0 ura3D0 kkq8Δ::URA3*	This paper
S. cerevisiae, BY4741 strain: YTM1023-1025,<br><br>*MATa his3D1 leu2D0 met15D0 ura3D0 bgl2Δ::URA3*	This paper
S. cerevisiae, BY4741 strain: YTM1028-1030,<br><br>*MATa his3D1 leu2D0 met15D0 ura3D0 pho85Δ::URA3*	This paper
S. cerevisiae, BY4741 strain: YTM1033-1035,<br><br>*MATa his3D1 leu2D0 met15D0 ura3D0 rad18Δ::URA3*	This paper
S. cerevisiae, BY4741 strain: YTM551,552<br><br>*MATa his3D1 leu2D0 met15D0 ura3D0 sic1Δ::URA3 YCplac111* (Colony (a))	This study
S. cerevisiae, BY4741 strain: YTM555,556<br><br>*MATa his3D1 leu2D0 met15D0 ura3D0 sic1Δ::URA3 YCplac111* (Colony (b))	This study
S. cerevisiae, BY4741 strain: YTM559,560<br><br>*MATa his3D1 leu2D0 met15D0 ura3D0 sic1Δ::URA3 YCplac111* (Colony (c))	This study
S. cerevisiae, BY4741 strain: YTM563,564<br><br>*MATa his3D1 leu2D0 met15D0 ura3D0 sic1Δ::URA3 YCplac111* (Colony (d))	This study
S. cerevisiae, BY4741 strain: YTM567,568<br><br>*MATa his3D1 leu2D0 met15D0 ura3D0 sic1Δ::URA3 YCplac111* (Colony (e))	This study
S. cerevisiae, BY4741 strain: YTM571,572<br><br>*MATa his3D1 leu2D0 met15D0 ura3D0 sic1Δ::URA3 YCplac111-SIC1-3HA* (Colony (a))	This study
S. cerevisiae, BY4741 strain: YTM575,576<br><br>*MATa his3D1 leu2D0 met15D0 ura3D0 sic1Δ::URA3 YCplac111-SIC1-3HA* (Colony (b))	This study
S. cerevisiae, BY4741 strain: YTM579,580<br><br>*MATa his3D1 leu2D0 met15D0 ura3D0 sic1Δ::URA3 YCplac111-SIC1-3HA* (Colony (c))	This study

S. cerevisiae, BY4741 strain: YTM583,584<br><br>*MATa his3D1 leu2D0 met15D0 ura3D0 sic1∆::URA3 YCplac111-SIC1-3HA* (Colony (d))	This study
S. cerevisiae, BY4741 strain: YTM587,588<br><br>*MATa his3D1 leu2D0 met15D0 ura3D0 sic1∆::URA3 YCplac111-SIC1-3HA* (Colony (e))	This study
S. cerevisiae, BY4741 strain: YTM765-767<br><br>*MATa his3D1 leu2D0 met15D0 ura3D0 YEplac181*	This study
S. cerevisiae, BY4741 strain: YTM769,770<br><br>*MATa his3D1 leu2D0 met15D0 ura3D0 YEplac181-GCD6*	This study
S. cerevisiae, BY4741 strain: YTM773,774<br><br>*MATa his3D1 leu2D0 met15D0 ura3D0 YEplac181-TCP1*	This study
S. cerevisiae, BY4741 strain: YTM777,778<br><br>*MATa his3D1 leu2D0 met15D0 ura3D0 YEplac181-UPC2*	This study
S. cerevisiae, BY4741 strain: YTM781,782<br><br>*MATa his3D1 leu2D0 met15D0 ura3D0 YEplac181-AHA1-YDR215C*	This study
S. cerevisiae, BY4741 strain: YTM785,786<br><br>*MATa his3D1 leu2D0 met15D0 ura3D0 YEplac181-ADR1*	This study
S. cerevisiae, BY4741 strain: YTM789,790<br><br>*MATa his3D1 leu2D0 met15D0 ura3D0 YEplac181-RAD9*	This study
S. cerevisiae, BY4741 strain: YTM793,794<br><br>*MATa his3D1 leu2D0 met15D0 ura3D0 YEplac181-SPR28*	This study
S. cerevisiae, BY4741 strain: YTM797,798<br><br>*MATa his3D1 leu2D0 met15D0 ura3D0 YEplac181-MFB1/YDR220C*	This study
S. cerevisiae, BY4741 strain: YTM801,802<br><br>*MATa his3D1 leu2D0 met15D0 ura3D0 YEplac181-GTB1/YDR220C*	This study
S. cerevisiae, BY4741 strain: YTM805,806<br><br>*MATa his3D1 leu2D0 met15D0 ura3D0 YEplac181-YDR222W*	This study
S. cerevisiae, BY4741 strain: YTM809,810<br><br>*MATa his3D1 leu2D0 met15D0 ura3D0 YEplac181-CRF1*	This study

S. cerevisiae, BY4741 strain: YTM813,814<br><br>*MATa his3D1 leu2D0 met15D0 ura3D0 YEplac181-HTB1/HTA1*	This study
S. cerevisiae, BY4741 strain: YTM817,818<br><br>*MATa his3D1 leu2D0 met15D0 ura3D0 YEplac181-ADK1*	This study
S. cerevisiae, BY4741 strain: YTM821-823<br><br>*MATa his3D1 leu2D0 met15D0 ura3D0 YEplac181-SIR4*	This study
S. cerevisiae, BY4741 strain: YTM825,826<br><br>*MATa his3D1 leu2D0 met15D0 ura3D0 YEplac181-PCF11*	This study
S. cerevisiae, BY4741 strain: YTM829,830<br><br>*MATa his3D1 leu2D0 met15D0 ura3D0 YEplac181-IVY1-COX20-YDR203W*	This study
S. cerevisiae, BY4741 strain: YTM833,834<br><br>*MATa his3D1 leu2D0 met15D0 ura3D0 YEplac181-HEM1*	This study
S. cerevisiae, BY4741 strain: YTM837,838<br><br>*MATa his3D1 leu2D0 met15D0 ura3D0 YEplac181-RTN1*	This study
S. cerevisiae, BY4741 strain: YTM841,842<br><br>*MATa his3D1 leu2D0 met15D0 ura3D0 YEplac181-LYS4*	This study
S. cerevisiae, BY4741 strain: YTM845,846<br><br>*MATa his3D1 leu2D0 met15D0 ura3D0 YEplac181-PRP42*	This study
S. cerevisiae, BY4741 strain: YTM849,850<br><br>*MATa his3D1 leu2D0 met15D0 ura3D0 YEplac181-FMN1*	This study
S. cerevisiae, BY4741 strain: YTM853,854<br><br>*MATa his3D1 leu2D0 met15D0 ura3D0 YEplac181-MRPL7*	This study
S. cerevisiae, BY4741 strain: YTM857,858<br><br>*MATa his3D1 leu2D0 met15D0 ura3D0 YEplac181-SEC26*	This study
S. cerevisiae, BY4741 strain: YTM861,862<br><br>*MATa his3D1 leu2D0 met15D0 ura3D0 YEplac181-YDR239C*	This study
S. cerevisiae, BY4741 strain: YTM865,866<br><br>*MATa his3D1 leu2D0 met15D0 ura3D0 YEplac181-SNU56-BUD26*	This study
S. cerevisiae, BY4741 strain: YTM869,870<br><br>*MATa his3D1 leu2D0 met15D0 ura3D0 YEplac181-AMD2*	This study

S. cerevisiae, BY4741 strain: YTM873,874<br><br>*MATa his3D1 leu2D0 met15D0 ura3D0 YEplac181-PRP28*	This study
S. cerevisiae, BY4741 strain: YTM877,878<br><br>*MATa his3D1 leu2D0 met15D0 ura3D0 YEplac181-PEX5*	This study
S. cerevisiae, BY4741 strain: YTM881,882<br><br>*MATa his3D1 leu2D0 met15D0 ura3D0 YEplac181-MNN10*	This study
S. cerevisiae, BY4741 strain: YTM885,886<br><br>*MATa his3D1 leu2D0 met15D0 ura3D0 YEplac181-TRS23YDR246W-A*	This study
S. cerevisiae, BY4741 strain: YTM889,890<br><br>*MATa his3D1 leu2D0 met15D0 ura3D0 YEplac181-VHS1*	This study
S. cerevisiae, BY4741 strain: YTM893,894<br><br>*MATa his3D1 leu2D0 met15D0 ura3D0 YEplac181-YDR248C*	This study
S. cerevisiae, BY4741 strain: YTM897,898<br><br>*MATa his3D1 leu2D0 met15D0 ura3D0 YEplac181-YDR249C-YDR250C*	This study
S. cerevisiae, BY4741 strain: YTM901,902<br><br>*MATa his3D1 leu2D0 met15D0 ura3D0 YEplac181-PAM1*	This study
S. cerevisiae, BY4741 strain: YTM905,906<br><br>*MATa his3D1 leu2D0 met15D0 ura3D0 YEplac181-BTT1*	This study
S. cerevisiae, BY4741 strain: YTM909,910<br><br>*MATa his3D1 leu2D0 met15D0 ura3D0 YEplac181-MET32*	This study
S. cerevisiae, BY4741 strain: YTM913,914<br><br>*MATa his3D1 leu2D0 met15D0 ura3D0 YEplac181-CHL4*	This study
S. cerevisiae, BY4741 strain: YTM917,918<br><br>*MATa his3D1 leu2D0 met15D0 ura3D0 YEplac181-RMD5*	This study
S. cerevisiae, BY4741 strain: YTM921,922<br><br>*MATa his3D1 leu2D0 met15D0 ura3D0 YEplac181-CTA1*	This study
S. cerevisiae, BY4741 strain: YTM925,926<br><br>*MATa his3D1 leu2D0 met15D0 ura3D0 YEplac181-RKM4*	This study
S. cerevisiae, BY4741 strain: YTM929,930<br><br>*MATa his3D1 leu2D0 met15D0 ura3D0 YEplac181-HSP78*	This study

S. cerevisiae, BY4741 strain: YTM933,934<br><br>*MATa his3D1 leu2D0 met15D0 ura3D0 YEplac181-YAP6*	This study
S. cerevisiae, BY4741 strain: YTM937,938<br><br>*MATa his3D1 leu2D0 met15D0 ura3D0 YEplac181-SWM1*	This study
S. cerevisiae, BY4741 strain: YTM941,942<br><br>*MATa his3D1 leu2D0 met15D0 ura3D0 YEplac181-EXG2*	This study
S. cerevisiae, BY4741 strain: YTM1003<br><br>*MATa his3D1 leu2D0 met15D0 ura3D0 sir4Δ::KanMX*	This study
S. cerevisiae, BY4741 strain: YTM1099-1106<br><br>*MATa his3D1 leu2D0 met15D0 ura3D0 sir4Δ::KanMX sic1Δ::URA3*	This study
S. cerevisiae, BY4741 strain: YTM552,553<br><br>*MATa his3D1 leu2D0 met15D0 ura3D0 sic1Δ::URA3 fob1Δ::natMX* (Colony (a))	This study
S. cerevisiae, BY4741 strain: YTM526,527<br><br>*MATa his3D1 leu2D0 met15D0 ura3D0 sic1Δ::URA3 fob1Δ::natMX* (Colony (b))	This study
S. cerevisiae, BY4741 strain: YTM530,531<br><br>*MATa his3D1 leu2D0 met15D0 ura3D0 sic1Δ::URA3 fob1Δ::natMX* (Colony (c))	This study
S. cerevisiae, BY4741 strain: YTM532,533<br><br>*MATa his3D1 leu2D0 met15D0 ura3D0 sic1Δ::URA3 fob1Δ::natMX* (Colony (d))	This study
S. cerevisiae, BY4741 strain: YTM535,536<br><br>*MATa his3D1 leu2D0 met15D0 ura3D0 sic1Δ::URA3 fob1Δ::natMX* (Colony (e))	This study
S. cerevisiae, BY4741 strain: YTM333-335<br><br>*MATa his3D1 leu2D0 met15D0 ura3D0 spt3Δ::kanMX*	This study
S. cerevisiae, BY4741 strain: YTM1204-1208, 1215-1219, 1520-1524<br><br>*MATa his3D1 leu2D0 met15D0 spt3Δ::kanMX sic1Δ::URA3*	This study

Table S2: Plasmids

Plasmid	source
YCplac111	Gietz and Sugino
YCplac111-SIC1-3HA	This paper
YEplac181	Gietz and Sugino
YEplac181-GCD6	This paper
YEplac181-TCP1	This paper
YEplac181-UPC2	This paper
YEplac181-AHA1-YDR215C	This paper
YEplac181-ADR1	This paper
YEplac181-RAD9	This paper
YEplac181-SPR28	This paper
YEplac181-MFB1/YDR220C	This paper
YEplac181-GTB1/YDR220C	This paper
YEplac181-YDR222W	This paper
YEplac181-CRF1	This paper
YEplac181-HTB1/HTA1	This paper
YEplac181-ADK1	This paper
YEplac181-SIR4	This paper
YEplac181-PCF11	This paper
YEplac181-IVY1-COX20-YDR203W	This paper
YEplac181-HEM1	This paper
YEplac181-RTN1	This paper
YEplac181-LYS4	This paper
YEplac181-PRP42	This paper
YEplac181-FMN1	This paper
YEplac181-MRPL7	This paper
YEplac181-SEC26	This paper
YEplac181-YDR239C	This paper

YEplac181-SNU56-BUD26	This paper
YEplac181-AMD2	This paper
YEplac181-PRP28	This paper
YEplac181-PEX5	This paper
YEplac181-MNN10	This paper
YEplac181-TRS23YDR246W-A	This paper
YEplac181-VHS1	This paper
YEplac181-YDR248C	This paper
YEplac181-YDR249C-YDR250C	This paper
YEplac181-PAM1	This paper
YEplac181-BTT1	This paper
YEplac181-MET32	This paper
YEplac181-CHL4	This paper
YEplac181-RMD5	This paper
YEplac181-CTA1	This paper
YEplac181-RKM4	This paper
YEplac181-HSP78	This paper
YEplac181-YAP6	This paper
YEplac181-SWM1	This paper
YEplac181-EXG2	This paper

Table S3: Oligonucleotides

Sequence	source
Primer for chr.XII probe template, HS204: CATTTCCTATAGTTAACAGGACATGCC	Hosoyamada et al.
Primer for chr.XII probe template, HS205: AATTCGCACTATCCAGCTGCACTC	Hosoyamada et al.
Primer for chr.IV probe template, oTM167: CTTGGCCACATCCAGAAATG	This paper
Primer for chr.IV probe template, oTM207: GGCAGCTGTGTAGAAATCACCAC	This paper
Primer for the region from the right side of Ty2-Ty1 to the telomere, oTM171: AAGTCGGAATTGACCAAACG	This paper
Primer for the region from the right side of Ty2-Ty1 to the telomere, oTM178: CCATATCATTCATATGCGAGG	This paper
Primer for IGS-F probe template, HS274: AGGAATATCGGAGGAGAATATTGTT	Hosoyamada et al.
Primer for IGS-F probe template, HS275: TTCTAGTTTCTTGGCTTCCTATGCT	Hosoyamada et al.
Primer for IGS-R probe template, HS266: TGCAAAGATGGGTTGAAAGAGAA	Hosoyamada et al.
Primer for IGS-R probe template, HS296: CCGCGTCGCCGCGTCGCCAAAAAT	Hosoyamada et al.
Primer for ACT1 probe template, HS270: CCGCGTCGCCGCGTCGCCAAAAAT	Hosoyamada et al.
Primer for ACT1 probe template, HS271: TGTCTTCCCATCTATCGTCGGTA	Hosoyamada et al.
Primer for SIR4 RT-qPCR oTM756: AGGGTTCATTGGAGCTTCAGTCA	This paper

Primer for SIR4 RT-qPCR oTM757:<br><br>AGGCTGATGCTTCGAAGGTG	This paper
Primer for IGS-F RT-qPCR oYY83:<br><br>CCCATAACTAACCTACCATTCGA	This paper
Primer for IGS-F RT-qPCR oYY84:<br><br>TCAAGTAGTAGCAACCCAATGAG	This paper
Primer for IGS-R RT-qPCR oYY85:<br><br>GGCTATTCATCTTGCACTTTTCC	This paper
Primer for IGS-R RT-qPCR oYY86:<br><br>GGCAGTTTCTAGGGAATGATGA	This paper
Primer for ACT1 RT-qPCR oYY34:<br><br>CGAATTGAGAGTTGCCCCAG	This paper
Primer for ACT1 RT-qPCR oYY35:<br><br>CAAGGACAAAACGGCTTGGA	This paper

Table S4: Antibodies

antibody	source	identifier
Mouse monoclonal anti-HA antibody, clone F-7	Santa Cruz Biotechnology	Cat# sc-7392
Sheep anti-mouse IgG HRP antibody	Cytiva	Cat# NA931-100UL
Rat anti-Yeast TUBULIN ALPHA HRP, clone YL1/2	Bio-Rad	Cat# MCA77P;RRID: AB_2021090
